# Sex-biasing influence of autism-associated *Ube3a* gene overdosage at connectomic, behavioral, and transcriptomic levels

**DOI:** 10.1126/sciadv.adg1421

**Published:** 2024-07-12

**Authors:** Caterina Montani, Luigi Balasco, Marco Pagani, Filomena Grazia Alvino, Noemi Barsotti, A. Elizabeth de Guzman, Alberto Galbusera, Alessia de Felice, Thomas K. Nickl-Jockschat, Sara Migliarini, Simona Casarosa, Pierre Lau, Lorenzo Mattioni, Massimo Pasqualetti, Giovanni Provenzano, Yuri Bozzi, Michael V. Lombardo, Alessandro Gozzi

**Affiliations:** ^1^ Functional Neuroimaging Laboratory, Istituto Italiano di Tecnologia, Center for Neuroscience and Cognitive Systems, CNCS@UNITN, Rovereto, Italy.; ^2^ Department of Cellular, Computational and Integrative Biology (CIBIO), University of Trento, Trento, Italy.; ^3^ Autism Center, Child Mind Institute, New York, NY, USA.; ^4^ IMT School for Advanced Studies, Lucca, Italy.; ^5^ Unit of Cell and Developmental Biology, Department of Biology, University of Pisa, Pisa, Italy.; ^6^ Department of Psychiatry and Psychotherapy, Otto-von-Guericke University, Magdeburg, Germany.; ^7^ German Center for Mental Health (DZPG), partner site Halle-Jena-Magdeburg, Germany.; ^8^ Center for Intervention and Research on adaptive and maladaptive brain Circuits underlying mental health (C-I-R-C), Halle-Jena-Magdeburg, Germany.; ^9^ Centre for Medical Sciences (CISMed), University of Trento, Trento, Italy.; ^10^ Istituto Italiano di Tecnologia, Center for Human Technologies, Genova, Italy.; ^11^ Center for Mind/Brain Sciences (CIMeC), University of Trento, Rovereto, Italy.; ^12^ CNR Neuroscience Institute, Pisa, Italy.; ^13^ Laboratory for Autism and Neurodevelopmental Disorders, Istituto Italiano di Tecnologia, Center for Neuroscience and Cognitive Systems, CNCS@UNITN, Rovereto, Italy.

## Abstract

Genomic mechanisms enhancing risk in males may contribute to sex bias in autism. The ubiquitin protein ligase E3A gene (*Ube3a*) affects cellular homeostasis via control of protein turnover and by acting as transcriptional coactivator with steroid hormone receptors. Overdosage of *Ube3a* via duplication or triplication of chromosomal region 15q11-13 causes 1 to 2% of autistic cases. Here, we test the hypothesis that increased dosage of *Ube3a* may influence autism-relevant phenotypes in a sex-biased manner. We show that mice with extra copies of Ube3a exhibit sex-biasing effects on brain connectomics and autism-relevant behaviors. These effects are associated with transcriptional dysregulation of autism-associated genes, as well as genes differentially expressed in 15q duplication and in autistic people. Increased Ube3a dosage also affects expression of genes on the X chromosome, genes influenced by sex steroid hormone, and genes sex-differentially regulated by transcription factors. These results suggest that *Ube3a* overdosage can contribute to sex bias in neurodevelopmental conditions via influence on sex-differential mechanisms.

## Teaser

Overdosage of autism risk gene *Ube3a* produces sex-dependent alterations in gene expression and brain connectivity.

## INTRODUCTION

Early-onset neurodevelopmental conditions tend to show a sex bias, with males being more affected than females (*1*). This imbalance is especially evident in the case of autism, where the male:female ratio is around 3:1 (*2*). Several ideas have been proposed to explain this phenomenon (*3*, *4*). Multiple risk factors that enhance risk in males have been identified, including the influence of steroid hormones, (*5*, *6*) and their prenatal programming effect on sex differences in structural and functional brain development of relevance to autism (*7*–*9*). Also, evidence for a possible genetic female protective effect has been reported. Autistic females tend to show an increased burden of rare de novo variants (*10*, *11*) as well as higher polygenic risk from inherited common variants (*12*, *13*). Rare deleterious variants are also transmitted maternally at higher rates (*14*, *15*). Both female protective and male risk factors have been theorized to work concurrently within a multiple liability threshold model of sex-differential risk for autism (*16*). However, despite these theoretical underpinnings, the exact mechanisms and genetic determinants that explain sex bias in autism are still largely unknown.

Sex-specific genetic, transcriptomic, and regulatory architectures are implicated in most diseases and complex traits (*17*–*19*). Together with sex-differential hormonal environments affecting mid-gestational periods (*7*, *20*), genetic risk factors may interact with sex to produce differential multiomic effects (e.g., at transcriptome, connectome, and phenome levels) that could either amplify risk in males or reduce risk in females, and thereby result in a sex bias in autism. A key mechanism that may exert such sex-differential multiomic effects in autism may reside within the function of the ubiquitin protein ligase E3A (Ube3a). *Ube3a* is located on chromosome 15q11-13, and deletions of this chromosomal region result in Prader-Willi or Angelman syndrome (AS), depending on whether the paternal or maternal copy is deleted (*21*). Duplication or triplication of this region also has an important neurodevelopmental impact, resulting in intellectual disability, epilepsy, and autism—a syndrome commonly referred to as dup15q syndrome (*22*–*24*). These genetic alterations can explain 1 to 2% of all autism cases and thus represent one of the strongest genetic risk factors for autism (*25*, *26*). In keeping with this, animal studies have shown that the increased *Ube3a* dosage reconstitutes autism-like traits in animals, an effect that may be mediated by impaired glutamatergic transmission (*27*).

Ube3a is commonly known for its role in protein degradation, and numerous proteins involved in neurodevelopment and autism have been reported to be a ubiquitination target of this protein, including TSC2 (*28*), Ephexin5 (*29*, *30*), SK2 (*31*), and XIAP (*32*). However, a less investigated independent function (*33*, *34*) through which Ube3a can affect brain development is its role as transcriptional coactivator with steroid hormone receptors (*35*). Steroid hormone receptors are known to affect developmental mechanisms related to autism (*7*, *36*–*39*) and thereby represent one possible mechanistic avenue for explaining sex bias in neurodevelopmental disorders. Through these functions, Ube3a can thus affect the transcriptomic and proteomic architecture of the developing brain (*40*–*42*) and may serve as a putative effector of sex-specific phenotypes of relevance to autism. Previous work supports the mechanistic plausibility of this framework, as gene expression analysis in predominantly male samples has shown convergence of cortical transcriptome dysregulation in idiopathic autism and dup15q syndrome (*43*).

Here, we test the hypothesis that increased dosage of *Ube3a* may exert a sex-biasing influence on autism-related phenotypes of high translational relevance. We used the Ube3a2X mouse model (*27*, *32*, *44*), mimicking maternally inherited 15q11-13 triplication, to investigate how such a genomic risk factor interacts with sex to produce differential effects at connectomic, behavioral, and transcriptomic levels, i.e., Ube3a2X mice harbor two extra-copies of *Ube3a* transgene and exhibit deficits in cortical excitatory transmission, together with core autism traits of relevance for dup15q syndrome (*27*, *44*). We found that Ube3a can critically contribute to sex bias via transcriptional influence on genes located on the X chromosome and downstream targets of the androgen receptor, including multiple high-confidence autism-associated genes. Our results uncover a powerful sex-biasing genomic influence of Ube3a that could explain some of the sex bias in autism and related neurodevelopmental disorders.

## RESULTS

### *Ube3a* gene dosage affects prefrontal and hypothalamic functional connectivity in a sex-specific dependent fashion

Robust alterations in brain anatomy and functional connectivity have been described in multiple autism mouse models, including mouse lines harboring genetic alterations associated with dup15q syndrome (*45*, *46*). The observed anatomical and functional alterations partly recapitulate abnormalities observed in patient populations and are thus considered a sensitive marker of developmental dysfunction. To investigate whether *Ube3a* dosage exerts sex-specific effects on brain circuits, we first carried out spatially unbiased resting-state functional magnetic resonance imaging (rsfMRI) connectivity mapping in male and female mice with increased *Ube3a* gene dosage (Ube3a2X), modeling dup15q syndrome (*27*).

**Fig. 1.**
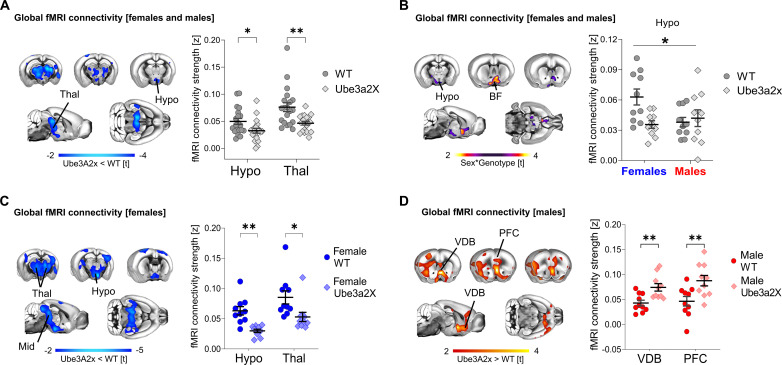
Increased *Ube3a* dosage affects global fMRI connectivity in a sex-dependent manner. (**A**) Contrast maps (left panel) illustrating difference in global fMRI connectivity strength between WT (*n* = 20) and Ube3a2X (*n* = 20) animals, irrespective of sex (blue indicates reduced connectivity, *t* test, *t* > 2; FWE cluster-corrected). Panel on the right illustrates quantification of global fMRI connectivity strength in representative regions of interest (*t* test; Hypothalamus, *t* = 2.44, *P* = 0.019; Thalamus, *t* = 3.36, *P* = 0.002). (**B**) Contrast maps (left panel) showing areas exhibiting sex*genotype interaction in global fMRI connectivity strength (purple-yellow indicates areas with significant interaction, *t* > 2; FWE cluster-corrected). Panel on the right illustrates the quantification of sex*genotype interaction in the hypothalamus (ANOVA, sex*genotype interaction, *F* = 5.85, *P* = 0.02). (**C**) Contrast maps (left panel) showing areas exhibiting decreased global fMRI connectivity strength in female Ube3a2X mice (*n* = 10) compared to female WT (*n* = 10) littermates (*t* test, *t* > 2; FWE cluster-corrected). The plot on the right illustrates quantification of global fMRI connectivity strength in representative regions of interest (*t* test; Hypothalamus, *t* = 4.50, *P* < 0.001; Thalamus, *t* = 2.44, *P* = 0.026). (**D**) Contrast maps (left panel) showing regions exhibiting increased global fMRI connectivity strength in male Ube3a2X mice (*n* = 10) compared to male WT (*n* = 10) littermates (red indicates increased connectivity, *t* test, *t* > 2; FWE cluster-corrected). Panel on the right illustrates quantification of global fMRI connectivity strength in both groups of males in representative regions of interest (*t* test; VDB, *t* = 3.33, *P* = 0.004; PFC, *t* = 2.90, *P* = 0.009). BF, basal forebrain; Hypo, hypothalamus; PFC, prefrontal cortex; Thal, thalamus; VDB, ventral diagonal band; Mid, Midbrain. *P < 0.05, **P < 0.01. Error bars indicate SEM.

Using weighted degree centrality as a metric of global connectivity (*47*), we identified foci of global hypoconnectivity in the hypothalamus and thalamus of Ube3a2X mice, irrespective of sex (*t* > |2.1|, cluster-corrected; Fig. 1A). Sex-specific effects were also found with significant sex*genotype interactions in the basal forebrain and hypothalamic regions (*t* > |2.1|, cluster-corrected; Fig. 1B). As shown in Fig. 1B, this interaction effect reflected reduced global connectivity in Ube3a2X females (*P* = 0.03; Fig. 1B). In contrast, Ube3a2X males displayed a nonsignificant trend for increased functional connectivity in both prefrontal and hypothalamic areas (*P* = 0.10; Fig. 1B).

**Fig. 2.**
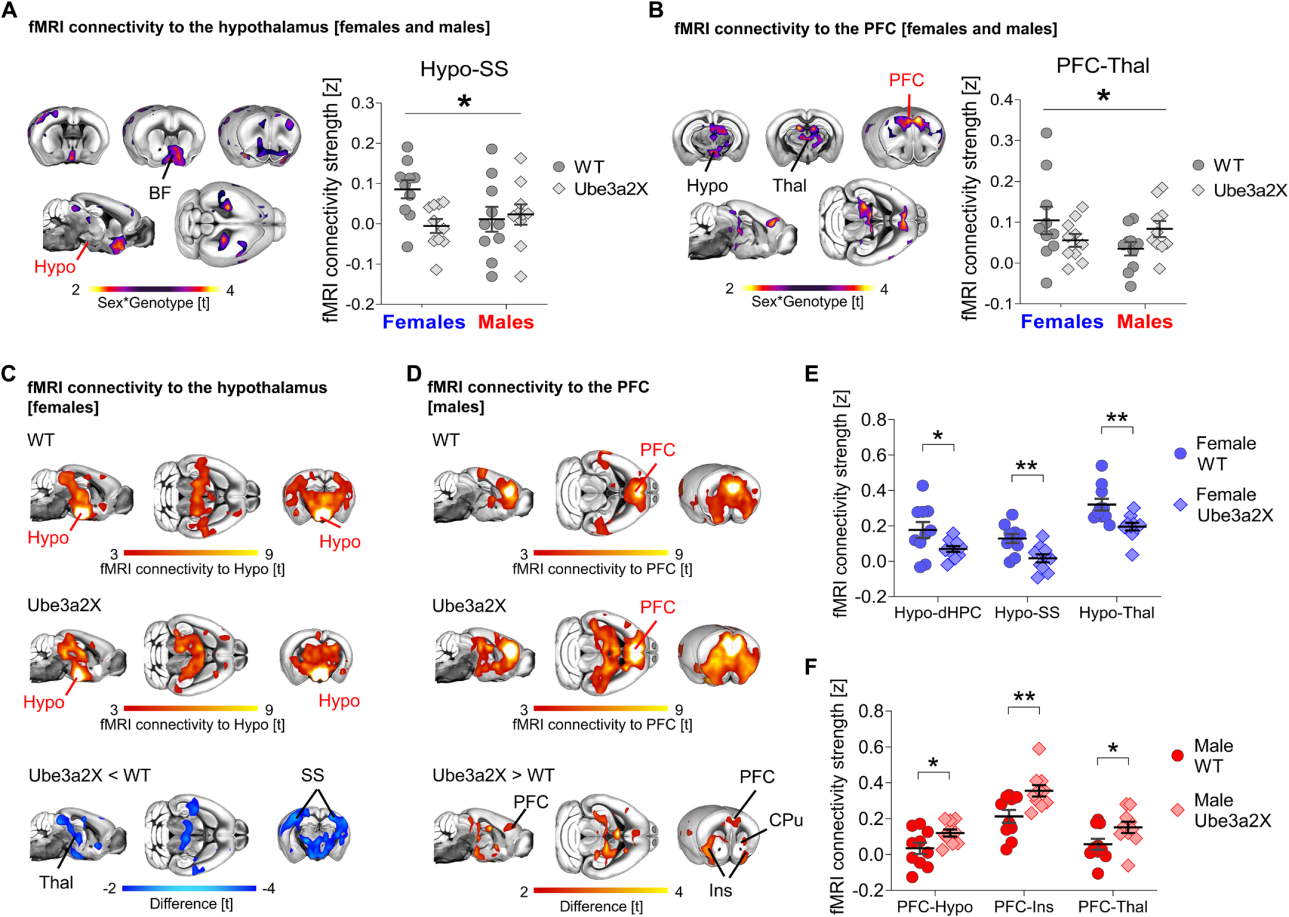
Divergent fMRI connectivity profiles in male and female Ube3a2X mutants. Seed-based connectivity mapping of (**A**) hypothalamus and (**B**) PFC. Contrast maps show areas exhibiting sex*genotype interaction of connectivity to the seed (purple-yellow coloring, sex*genotype interaction, *t* > 2; FWE cluster-corrected). The plots on the right illustrate the quantification of sex*genotype interaction of connectivity strength between the seed and the region of interest (ANOVA, Hypo-SS *F* = 4.42, *P* = 0.04, PFC-Thal *F* = 4.62, *P* = 0.04). Seed-based connectivity mapping of (**C**) hypothalamus in WT and Ube3a2X female mice, and (**D**) PFC in male WT and Ube3a2X mutants. Red-yellow coloring represents regions exhibiting fMRI connectivity with the seed region in control and Ube3a2X mice (WT, top panels; Ube3a2X, middle panels; one-sample *t* test, *t* > 3). Contrast maps are at the bottom of the panel (blue indicates reduced connectivity in Ube3a2X females, red indicates increased connectivity in Ube3a2X males, *t* test, *t* > 2). All statistics are FWE cluster-corrected. Quantification of connectivity strength between seeds and region of interest in the (**E**) female and (**F**) male groups (*P < 0.05, **P < 0.01, unpaired *t* test). Seed regions are indicated in red lettering. BF, basal forebrain; Hypo, hypothalamus; PFC, prefrontal cortex; Thal, thalamus; dHPC, dorsal hippocampus; Ins, insula; SS, somatosensory cortex; CPu, Caudate Putamen. *P < 0.05, **P < 0.01. Error bars indicate SEM, and each dot represents a mouse. Ube3a2X, *n* = 20 versus WT *n* = 20, *n* = 10 males and females within each group.

Given the presence of sex*genotype interactions, we next carried out follow-up analyses in male and female mice, separately. These analyses revealed reduced global connectivity across a large set of mid-brain, hypothalamic, thalamic, and sensory areas in Ube3a2X females (*t* > |2.1|, cluster-corrected; Fig. 1C). The observed connectivity changes in Ube3a2X female mice are suggestive of a potential reduction in sex dimorphism in mutants compared to control animals. In contrast, Ube3a2X males showed increased global connectivity in prefrontal and basal forebrain areas (*t* > |2.1|, cluster-corrected; Fig. 1D). To probe the circuit-level substrates differentially affected in the two sexes, we next performed a set of seed-based connectivity analyses in regions exhibiting global connectivity differences (Fig. 2). These investigations revealed foci with significant sex*genotype interactions in hypothalamic, basal forebrain, and medial prefrontal areas, corroborating the involvement of these areas as key substrates for sex-divergent functional dysconnectivity produced by increased *Ube3a* dosage [*t* > |2.1|, family-wise error rate (FWER) cluster-corrected; Fig. 2, A and B]. Further investigation of these effects in each sex separately (Fig. 2, C to F) revealed that in Ube3a2X females, hypothalamic areas exhibit prominent hypoconnectivity with somatosensory cortex, thalamus, and hippocampus (*t* > |2.1|, FWER cluster-corrected; Fig. 2, C and E). In contrast, Ube3a2X males were characterized by functional hyperconnectivity between the medial prefrontal cortex (PFC), the anterior insula, and thalamic and hypothalamic regions (*t* > |2.1|, FWER cluster-corrected; Fig. 2, D and F). These findings suggest that hypothalamic and prefrontal circuits exhibit sex-specific, divergent patterns of dysconnectivity in mice with increased dosage of *Ube3a*.

Local fMRI connectivity is also often disrupted in mouse models of autism (*47*, *48*). We thus investigated if sex-biased changes in connectivity would also be detectable on a local scale. Local connectivity mapping in Ube3a2X mice revealed foci of robustly decreased local connectivity in hypothalamus, thalamus, and hippocampus (*t* > |2.1|, cluster-corrected; fig. S1A), irrespective of sex. Significant sex*genotype interactions were observed in prefrontal, hippocampal, and hypothalamic regions (*t* > |2.1|, cluster-corrected; fig. S1B). This effect was mainly driven by decreased local connectivity in Ube3a2X females (*P* = 0.04; fig. S1B). Sex-specific effects were not apparent in brain anatomy (fig. S2). Gray matter (GM) voxel-based morphometry (*49*) revealed robust bilateral reductions in GM volume in the amygdala, thalamus, and hippocampus in Ube3a2X mice irrespective of sex (*t* > |2|, cluster-corrected; fig. S2). No sex*genotype interactions were identified upon voxelwise mapping (*t* > |2.1|; fig. S2C). Together, these imaging studies show that hypothalamic and prefrontal circuits exhibit divergent, sex-specific patterns of functional dysconnectivity in Ube3a2X mice.

### Male Ube3a2X mice exhibit increased stereotyped behavior

**Fig. 3.**
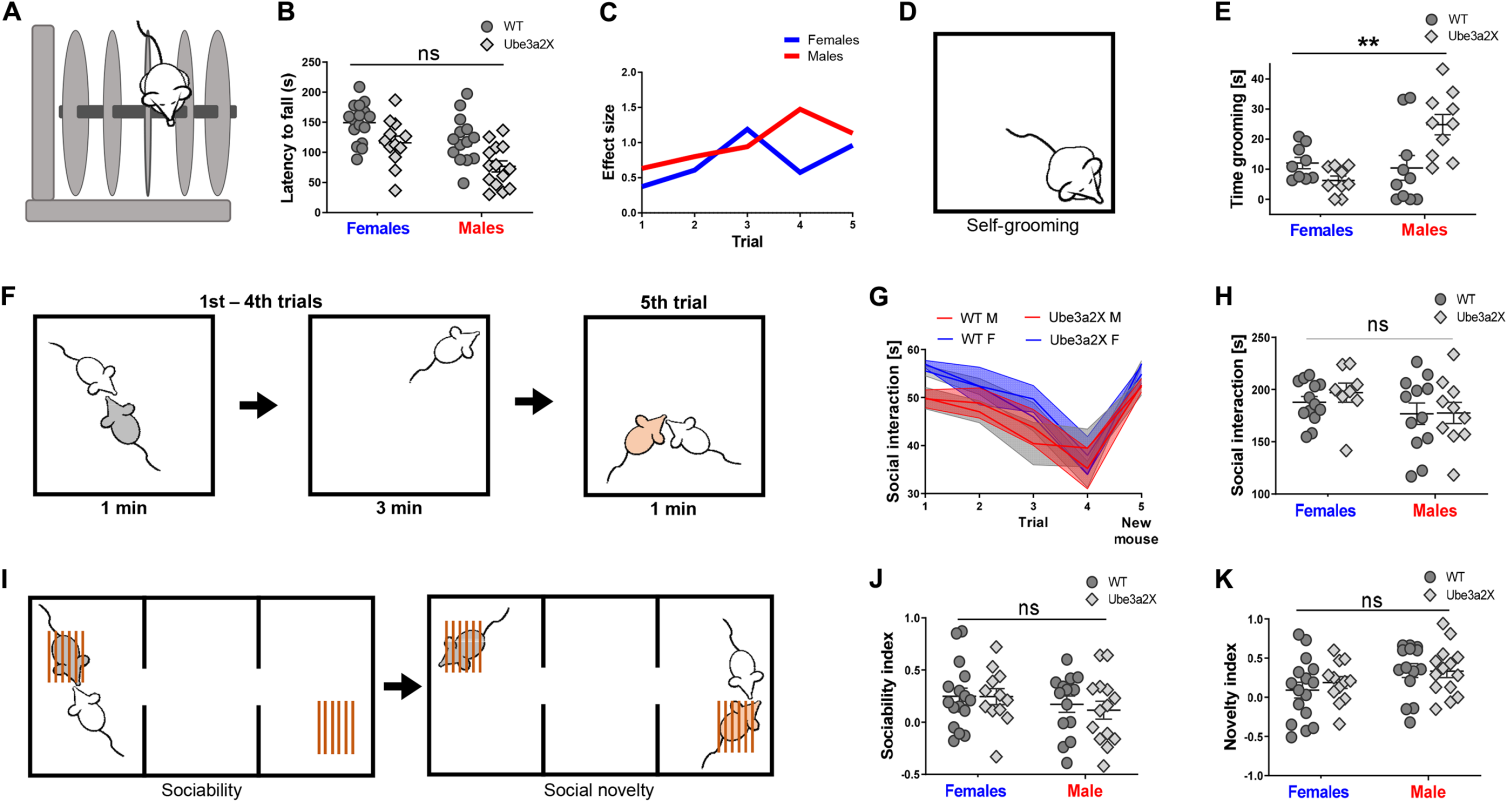
* Ube3a* dosage affects stereotyped behavior in a sex-dependent manner. (**A**) Rotarod test to assess locomotor activity. (**B**) Quantification of latency to fall (Ube3a2X *n* = 26, *n* = 14 males and *n* = 12 females; WT *n* = 30, *n* = 14 males and *n* = 16 females). Sex*genotype interaction was not significant (ANOVA, *F* = 0.29, *P* = 0.59). Both sex and genotype factors were instead significant (*F* = 12.3, *P* < 0.001 and *F* = 16.2, *P* < 0.001, respectively), driven by decreased latency in transgenic males (Tukey’s post hoc test, ***P < 0.001). (**C**) Cohen’s d effect size for the latency to fall. (**D**) Schematics of the self-grooming test. (**E**) Quantification of time spent grooming (Ube3a2X *n* = 20, *n* = 10 males and females; WT *n* = 20, *n* = 10 males and females). Sex*genotype interaction was significant (ANOVA, *F* = 10.95, **P = 0.002) and driven by increased grooming in male mutants. (**F**) Habituation/dishabituation social interaction test. (**G**) Social interaction duration in the habituation/dishabituation test for all trials (WT *n* = 25, *n* = 12 males and *n* = 13 females; Ube3a2X *n* = 18, *n* = 10 males and *n* = 8 females). (**H**) Cumulative social interaction during the first four trials of the habituation/dishabituation test. Sex*genotype interaction was not significant (ANOVA, *F* = 0.21, *P* = 0.64). (**I**) Schematics of the three-chamber test. (**J**) Quantification of the sociability index (WT *n* = 30, *n* = 14 males and *n* = 16 females; Ube3a2X *n* = 26, *n* = 14 males and *n* = 12 females). Sex*genotype interaction was not significant (ANOVA, *F* = 0.11, *P* = 0.73). (**K**) Quantification of novelty index. Sex*genotype interaction was not significant (ANOVA, *F* = 0.33, *P* = 0.57).

The observation of sex*genotype interactions in fMRI connectivity led us to investigate whether sex-specific behavioral dysfunction would be detectable in behavioral domains relevant to autism and other male-biased neurodevelopmental disorders. Motor issues (e.g., delays in achieving early motor milestones, hypotonia, clumsiness, and difficulties across visuomotor, fine, and gross motor skills) are a common, yet nondiagnostic, feature of many autistic individuals that increases with increased severity in core diagnostic domains (*50*–*52*). Previous research also indicates that 15qdup syndrome in humans is associated with motor impairments (*53*, *54*). We thus used the rotarod test to probe the presence of sex*genotype interactions in locomotor activity and motor coordination in Ube3a2X mice (Fig. 3). We found that Ube3a2X mutants exhibited motor impairments as assessed with latency to fall score [*F* = 16.2; *P* < 0.001, genotype, two-way analysis of variance (ANOVA); Fig. 3B], but this effect was not sex-specific (sex*genotype interaction, *F* = 0.29, *P* = 0.59; Fig. 3C). Further investigations using the open-field test (fig. S3A) revealed that Ube3a2X mice, irrespective of sex, showed comparable mobility (total distance traveled and frequency of rotations), time spent in the center of the field, and time spent wall rearing to control wild-type (WT) littermates (*P* > 0.32, genotype, all tests, two-way ANOVA; fig. S3A). These results rule out the presence of prominent anxiety-like phenotypes or hyperactivity in these mutants, hence arguing against a confounding contribution of motor hyperactivity on the results obtained with the rotarod test.

We next investigated the presence of autism-like stereotyped behavior using self-grooming scoring (*55*). These investigations revealed robust sex*genotype interactions (*F* = 11.0; *P* = 0.002, two-way ANOVA; Fig. 3E), explained by Ube3a2X males spending more time self-grooming compared to WT male littermates (*P* = 0.008; Fig. 3E), whereas Ube3a2X females exhibited a reverse trend of less time spent on self-grooming (*P* = 0.56; Fig. 3E). These results show that increased stereotyped behaviors are present in male but not female mutant mice.

We next probed social behavior in control and Ube3a2X mutants using a habituation/dishabituation social interaction test (Fig. 3, F to H) (*56*). We did not observe any genotype-dependent difference in sociability in Ube3a2X mice, nor sex*genotype interactions, both in terms of interaction time with the familiar mouse and upon measuring interaction with a novel stimulus mouse (*F* = 0.78, *P* > 0.51, all comparisons, two-way ANOVA; Fig. 3, G and H). To further investigate social behavior in Ube3a2X mice, we also tested mutant and control mice in a three-chamber test (Fig. 3, I to K, and fig. S3B) (*55*). Also in this test, we did not find any significant genotype-dependent effects or sex*genotype interaction in either sociability (*F* = 0.11; *P* = 0.73; two-way ANOVA; Fig. 3J) or social novelty index (*F* = 0.33; *P* = 0.57; Fig. 3K). In summary, increased *Ube3a* dosage affects motor ability but not sociability or social habituation responses. Sex-specific effects on stereotyped behavior were apparent, indicative of autism-like increased stereotyped behaviors in male but not in female Ube3a2X mutants.

### Increased *Ube3a* dosage results in sex-specific PFC transcriptomic dysregulation

**Fig. 4.**
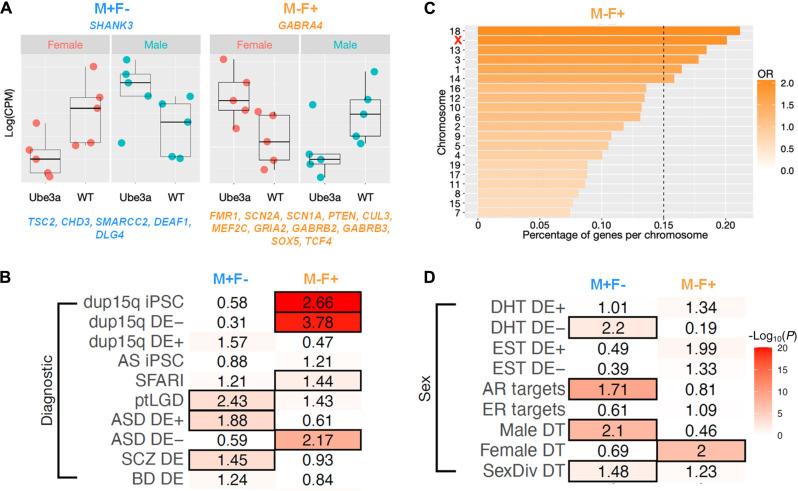
Sex-specific PFC transcriptomic dysregulation by *Ube3a* overexpression and enrichment with autism-associated, dup15q, and sex-relevant mechanisms. (**A**) Plots display log(CPM) for shank3 and gabra4, two examples of DE genes for the sex*genotype interaction (M+F−, light blue; M−F+, orange). High-confidence SFARI genes that belong to each of the two groups are at the bottom. (**B**) Heatmap showing enrichments with gene lists from dup15q (dup15q DE) (*43*), SFARI genes, private (pt) inherited likely gene disrupting (LGD) variants (*58*), autism spectrum disorders (ASD), schizophrenia (SCZ) and bipolar disorder (BD) (*59*), iPSC-derived neurons from dup15q (dup15q iPSC), and Angelman syndrome (AS iPSC) individuals (*60*). The symbols on the acronyms indicate down-regulated (minus) or up-regulated (plus) expression. (**C**) Plot showing the percentage of genes per each chromosome that are DE−. Color indicates the enrichment OR. The X chromosome is in red. The vertical dotted line indicates the FDR threshold. (**D**) Heatmap showing enrichments between sex*genotype interaction DE genes and genes relevant to sex hormones or sex-differential gene regulation. DHT DE+ and DHT DE− are genes that are up-regulated (plus) or down-regulated (minus) after dihydrotestosterone (DHT) manipulation (*7*). EST DE+ and EST DE− are genes that are up-regulated (plus) or down-regulated (minus) after treatment with estrogen (EST) (*115*). AR Targets are downstream target genes of the androgen receptor (AR) as defined by chromatin immunoprecipitation sequencing in human neural stem cells (*36*). ER Targets are downstream target genes of the estrogen receptor (ER) (*37*). Male DT, Female DT, and SexDiv DT genes are sex-differentially targeted by transcription factors (*116*). The numbers in each cell indicate the enrichment OR, and the color indicates the–log_10_(*P* value) for the enrichment test. Cells outlined in black pass FDR. FDR *q* < 0.05 threshold for multiple comparisons correction.

Given the global and local connectivity abnormalities converging on the medial PFC and hypothalamus (Hypo), we next investigated if increased *Ube3a* dosage results in sex-specific transcriptomic dysregulation in those regions. Bulk tissue from the PFC and Hypo was used to quantify gene expression with RNA sequencing (RNA-seq), and analysis was tailored to identify differentially expressed (DE) genes for main effects of sex, genotype, and the sex*genotype interaction (tables S1 to S3). In the PFC, 2625 genes were detected as DE at false discovery rate (FDR) *q* < 0.05 for the sex*genotype interaction. In contrast, no genes survived FDR correction for the interaction effect in Hypo (fig. S4). PFC sex*genotype interaction DE genes fell into two main classes: (i) so-called “M−F+” genes, down-regulated in Ube3a2X males (e.g., WT > Ube3a2X) and up-regulated in Ube3a2X females, and (ii) so-called “M+F−” genes, up-regulated in Ube3a2X males (e.g., Ube3a2X > WT) and down-regulated in Ube3a2X females (Fig. 4A). For a list of genes that survived statistical thresholding of the main effect of sex, please see fig. S4 and table S2. The main effect of genotype identified only *Ube3a* after FDR correction in both PFC and Hypo (table S3). Control quantitative reverse transcription polymerase chain reaction (qRT-PCR) and Western blot analyses of Ube3a in an independent cohort of animals revealed comparable fronto-cortical mRNA and protein levels in male and female Ube3a2X mutants (fig. S5, A to D; *P* > 0.2, all comparisons). This result suggests that the observed sex-specific results did not trivially reflect different levels of Ube3a mRNA or protein expression in male and female Ube3a2X mutants.

To further corroborate a sex-biasing effect of *Ube3a* overdosage at the transcriptomic level, we first validated four RNA-seq top-hit genes via qRT-PCR in a separate cohort of mice. All four probed genes (*cul3*, *fmri1*, *gabrb3*, and *scn2a*) showed significant sex*genotype interaction in the PFC of our experimental mice, mirroring previous RNA-seq findings (fig. S5, E to H; ANOVA, sex*genotype interaction, *P* < 0.39, sex*genotype interaction, all genes). We next carried out a proof-of-concept experiment where Ube3a was perinatally overexpressed in male and female WT FVB mice at postnatal day 1 via intracerebroventricular (ICV) injection of the AAV-PHP.B-hSyn-hUBE3At vector. This construct has been previously used to enable in vivo overexpression of human Ube3a in the mouse brain (*57*). The goal of this investigation was to probe whether sex-biasing effects of Ube3a overdosage could be obtained in a different, nonconstitutive mouse model.

qRT-PCR quantifications of human Ube3a (*hUbe3a*) mRNA in the PFC of FVB mice at postnatal day 60 revealed abundant overexpression of this gene in both male and female mice (fig. S6, A and B). Despite the use of comparable amount of virus in both sexes, we found that *hUbe3a* was slightly more abundantly expressed in female mice (fig. S6B). Notwithstanding this difference, qRT-PCR quantification of four top-hit genes that are DE in Ube3a2X mice (i.e., *fmr1*, *cul3*, *gabrb3*, and *scn2a*) exhibited a sex-dependent expression that closely recapitulated the distribution observed in the constitutive (Ube3a2X) mouse model (fig. S6C). These results show that perinatal human *Ube3a* overexpression may produce sex-specific gene expression dysregulations comparable to what observed in the genetic model, thus corroborating the generalizability of our findings.

### Ube3a2X mouse DE gene enrichment for autism-associated and dup15q genes translates to humans

Having identified important sex-specific transcriptome dysregulation in PFC, we next asked if such genes are of relevance to known genetic mechanisms of importance in human patients with either autism or dup15q syndrome. The combined set of all M−F+ and M+F− genes was significantly enriched for genes annotated on SFARI Gene (https://gene.sfari.org) as being associated with autism [odds ratio (OR) = 1.57, *P* = 0.01]. This enrichment comprised many notable high-confidence genes such as *fmr1*, *shank3*, *scn2a*, *scn1a*, *pten*, *cul3*, *tsc2*, *mef2c*, *gria2*, *gabrb2*, *gabrb3*, *chd3*, *sox5*, *smarcc2*, *deaf1*, *dlg4*, and *tcf4.* Splitting the enrichment analysis in M+F− and M−F+ gene sets further revealed that this SFARI enrichment was driven primarily by the M−F+ gene set (Fig. 4B). Going beyond evidence in SFARI Gene, we also tested for enrichments with ultra-rare private inherited mutations (ptLGD) that contribute to at least 4.5% of autism risk (*58*). We found that M+F−, but not M−F+, genes were enriched for these ptLGD genes (Fig. 4B). In line with these results, we also found that M−F+ genes, which are down-regulated in Ube3a2X males, were enriched for genes that are down-regulated in postmortem cortical tissue of a primarily male sample of human patients with autism (*59*). In contrast, M+F− genes (i.e., up-regulated in Ube3a2X males) were enriched for genes with up-regulated expression in postmortem cortical tissue of a predominantly male group of patients with autism (Fig. 4B) (*59*). M+F− genes also overlapped with dysregulated cortical transcriptome signal in patients with schizophrenia (Fig. 4B), a finding that may be expected given some overlap in the genomic mechanisms involved in autism and schizophrenia. Further underscoring the mouse-human cross-species translational value of our findings, we found that M−F+ genes were highly enriched for DE genes in induced pluripotent stem cell (iPSC)–derived neurons from dup15q but not from AS individuals (Fig. 4B) (*60*). Furthermore, M−F+ genes were highly enriched for genes that are down-regulated in cortical tissue of human dup15q patients (Fig. 4B) (*43*). For a complete statistic of each gene list used in the enrichment tests, please see table S4. The background list and the complete gene list for each dataset are reported in tables S5 and S6, respectively. These enrichment results support the translational relevance for human patients with autism or dup15q syndrome of the sex-specific transcriptomic dysregulation produced by increased dosage of *Ube3a*.

### Sex-specific transcriptomic dysregulation of Ube3a affects sex-relevant genomic mechanisms

Sex differences in the brain are theorized to be mediated by mechanisms driven by genes located on the sex chromosomes—in particular on the X chromosome (*61*). Thus, we next tested whether PFC sex*genotype interaction genes were disproportionately more common on specific chromosomes such as the X chromosome, than expected by chance. We found that M−F+, but not M+F−, genes were disproportionately located on several chromosomes (significant after FDR *q* < 0.05) and that the X chromosome was one of these (Fig. 4C and fig. S7).

One of the prominent roles of Ube3a is its function as transcriptional coactivator with steroid hormone receptors (e.g., AR, ESR1, ESR2, and PGR). This suggests that Ube3a may influence transcription in a manner dependent on these sex-relevant mechanisms. To examine this hypothesis in more detail, we next investigated how PFC sex*genotype DE genes might overlap with gene lists incorporating sex-relevant mechanisms, such as genes sensitive to the transcriptional influence of sex steroid hormones, downstream targets of the androgen and estrogen receptors, or genes that are sex-differentially regulated by transcription factors. These analyses revealed that M+F− genes overlap significantly with downstream targets of the androgen receptor (AR Targets), but not targets of the estrogen receptor (ER Targets). M+F− genes were also enriched for genes down-regulated by potent androgens such as dihydrotestosterone (DHT DE−). Genes that are sex-differentially regulated by transcription factors were important as well. The M+F− set was enriched for genes that show stronger male-regulatory influence (Male DT) and for genes with relatively equal proportions of male-biased and female-biased transcription factors exerting regulatory influence (SexDiv DT). In contrast, M−F+ genes were significantly enriched only for genes with female-biased regulatory influence (Female DT) (Fig. 4D).

For a complete statistics of each gene list used in the enrichment tests, please see table S4. The background list and the complete gene list for each dataset are reported in tables S5 and S6, respectively. Overall, these results show that *Ube3a* overexpression affects gene networks and systems under the influence of diverse sex-relevant mechanisms, including the effect of genes sensitive to steroid hormone influence, downstream targets of steroid hormone receptors, as well as genes that are sex-differentially targeted by transcription factors.

### Sex-specific transcriptomic dysregulation by Ube3a affects convergent ASD-relevant biological systems and pathways

Several studies have noted common downstream biological processes/pathways and cell types that may unify the heterogeneous genomic and molecular basis behind autism spectrum disorder (ASD). Among the most important processes/pathways are synapse, transcription and chromatin remodeling, protein synthesis and translation, protein degradation, cytoskeleton processes, splicing, and numerous signaling pathways [e.g., RAS/extracellular signal–regulated kinase (ERK)/mitogen-activated protein kinase (MAPK), phosphatidylinositol 3-kinase (PI3K)/AKT/mammalian target of rapamycin (mTOR), and WNT) (*62*–*64*). Thus, we next examined the PFC sex*genotype DE gene sets for enrichments in these processes/pathways and cell types. For biological process enrichment analysis, we used GeneWalk to get context-specific and gene-level enrichments for Gene Ontology Biological Process (GO BP) terms. This analysis resulted in a variety of key ASD-relevant processes.

**Fig. 5.**
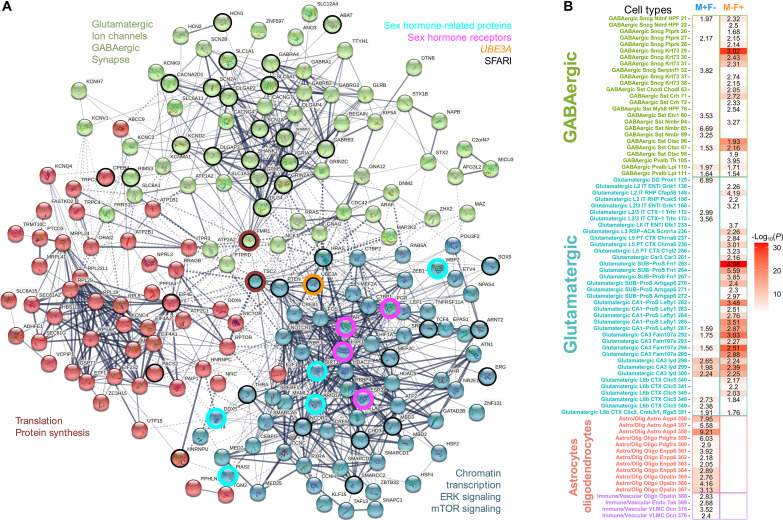
Sex-specific PFC transcriptomic dysregulation by Ube3a overexpression affects convergent ASD-relevant biological systems, pathways, and cell types. (**A**) PPI graph of PFC sex-by-genotype interaction genes. Nodes are colored according to a *k*-means clustering solution with *k* = 3. These clusters also segregate genes with GO BP enrichment terms specified in text next to each cluster. Nodes are circled in black if they are SFARI ASD genes. Nodes circled in turquoise are sex hormone–related proteins, whereas magenta circled nodes are sex hormone receptors. Ube3a is circled in orange. Fmr1 and Tsc2 are circled in red to indicate that these two translation and protein synthesis relevant genes also serve as gene connector hubs in the identified transcriptional network. (**B**) Cell type enrichment heatmap showing how M+F− or M−F+ PFC sex-by-genotype interaction gene sets (columns) are enriched in numerous cell type markers (specified on the rows) from the Allen Institute mouse scRNA-seq data (*65*). M−F+ genes strongly hit a variety of glutamatergic and GABAergic cell types, whereas M+F− genes show specific enrichments with astrocyte and oligodendrocyte cell types. The numbers in each cell indicate the enrichment OR, while the coloring indicates the–log_10_(*P* value). Only enrichments significant at FDR *q* < 0.05 are shown.

To visualize these GO BP processes and the DE genes that go along with such enrichments within protein-protein interaction (PPI) networks, we report an interaction graph in Fig. 5A. This illustrative plot provides a purely descriptive representation of a subset of the genes, which map onto three clusters of terms: (i) synaptic, glutamatergic, GABAergic, ion channel proteins (green); (ii) transcription and chromatin remodeling proteins, proteins within mTOR and ERK signaling pathways, as well as steroid hormone receptors (AR, ESR1, ESR2, PGR) and proteins that show enrichments for androgen and estrogen receptor signaling (blue); and (iii) proteins involved in translation and protein synthesis. This evidence of highly significant PPIs (actual edges = 1015, expected edges = 405, *P* < 1.0 × 10^−16^) and highly ASD-relevant GO BP enrichment showcases a clear example of how the PFC sex*genotype DE genes are embedded within a complex systems-level biological pathology that integrates abnormalities along these key processes and pathways.

### Sex-specific PFC transcriptomic dysregulation by Ube3a differentially affects neuronal and glia cell types

Finally, we asked what cell type markers are enriched within the PFC sex*genotype interaction gene set. Here, we used lists of cell type markers from a mouse single-cell transcriptomic atlas from the Allen Institute covering a diverse array of multiple glutamatergic and GABAergic neuronal cell types in mouse isocortex and hippocampus, as well as numerous glial and other nonneuronal cell types (*65*). These analyses uncovered that both M−F+ and M+F− gene sets were significantly enriched for a number of glutamatergic and GABAergic cell type markers. However, the magnitude and coverage of enrichments with these neuronal cell types were much stronger and broader for the M−F+ gene set. Setting the M+F− gene set apart from M−F+ genes, we also identified strong enrichments with numerous astrocyte and oligodendrocyte cell type markers, whereas no significant enrichments in these markers were present in the M−F+ gene set (Fig. 5B). This result is suggestive of cell type specificity in how *Ube3a* overexpression drives sex-specific PFC transcriptomic dysregulation. Genes down-regulated in males, but up-regulated in females, affected diverse glutamatergic and GABAergic cell types, whereas genes up-regulated in males but down-regulated in females affected astrocyte, oligodendrocyte, and some glutamatergic and GABAergic neuronal cell types.

## DISCUSSION

While the role of *Ube3a* mutations in determining monogenic forms of developmental disorders is well established (*66*), the possibility that *Ube3a* overdosage might exert sex-biasing influence has not been extensively explored. Here, we document a previously unreported sex-biasing effect of *Ube3a* gene overdosing at the connectomic, behavioral, and transcriptomic levels.

Our investigations revealed a sex-dependent effect of *Ube3a* overdosage on multiple translational (endo)phenotypes of relevance to autism, including changes in rsfMRI connectivity. Altered interareal rsfMRI coupling is a hallmark of autism, where the observed connectivity changes are prominent but also highly heterogeneous (*67*–*69*). Recently, fMRI-based connectivity mapping across 16 mouse mutants harboring different autism-relevant etiologies revealed a broad spectrum of connectional alteration (*70*), providing compelling evidence that heterogeneous findings in autism are likely to, at least partly, reflect the etiological heterogeneity of autism. Our results show that autism-relevant connectivity changes can also critically differ across sexes within the same etiological domain, hence underscoring a key, yet still underappreciated, dimension to the investigation of the origin and significance of functional dysconnectivity in brain disorders (*71*–*73*). More broadly, these findings add to the emerging concept that autism and related neurodevelopmental disorders are characterized by a broad spectrum of connectivity alterations that are very sensitive to (and strongly biased by) the underlying etiological mechanisms (*70*).

Self-grooming is an innate behavior of high translational relevance, as it is thought to recapitulate in rodents aberrant, stereotypical motor patterns that characterize several human disorders, including autism. The complex and distributed neural circuits implicated in different aspects of self-grooming in rodents have been recently reviewed (*74*) and include fronto-striatal networks, hypothalamus, thalamus, and amygdala. In this respect, the presence of fronto-striatal hyperconnectivity in Ube3a2X males is of interest, as it represents a network dysfunction that is commonly observed in multiple autism models (*70*) and that we found to be associated with increased grooming activity in other models of autism [i.e., Tsc2-deficient mice, (*75*)]. In the same study, we also showed that similar patterns of fronto-striatal hyperconnectivity are detectable in subsets of idiopathic autism patients, where they are associated with a gene coexpression network involving mTOR-Tsc2. These findings suggest that, while etiological diversity is a prominent contributor to autism heterogeneity, congruent autism relevant circuit dysfunction may arise as a result of the dysregulation of distinct, mechanistically dissociable, gene coexpression networks. This observation also underscores the possibility of translating behaviorally relevant network-level changes across species via the use of cross-species rsfMRI (*73*).

Our behavioral testing revealed that Ube3a2X mice exhibit robust motor deficits but failed to reproduce the social alterations previously described by Smith *et al.* (*27*). Other mouse models overexpressing Ube3a have been reported to have different behavioral profiles. For example, Ube3a overexpression in Camk2a-positive neurons (*45*) resulted in anxiety-like behaviors, learning impairments, and reduced seizure thresholds (but not social deficits or repetitive behaviors). A syntenic 6.3-Mb duplication of the mouse region orthologous to the human 15q11-13 region (including *Ube3a*) resulted instead in social and cognitive impairments (*76*). Finally, an additional model of *Ube3a* overdosage was very recently reported not to present any major alterations in social and cognitive behavior nor transcriptional changes, when these were probed at a whole-cortical level in the early postnatal phase (*77*). These investigators, however, described the presence of motor alterations in mice exhibiting a threefold protein Ube3a overdosage like in Ube3a2X mice. The fact that none of the studies rigorously examined the presence of sex*genotype interactions prevents a direct comparison with our results. Moreover, subtle experimental discrepancies, including differences in the genetic background and constructs used in these models (*12*), as well as in the testing parameters used in behavioral tests (*5*) may account for the discrepant phenotypic profile of these models. Future comparative investigations across mouse lines are warranted to identify and differentiate core versus ancillary phenotypes produced by Ube3a overdosing in mice.

Our work also reports a sex-invariant effect of Ube3a2X overdosage on large-scale neuroanatomy. Specifically, we report reduced GM volume in the amygdala, thalamus, and hippocampus of Ube3a2X mutants. Similar findings have been previously shown in mice overexpressing Ube3a in Camk2a-positive excitatory neurons (*45*). Dup15q patients have been reported to present brain morphological abnormalities, mostly involving the hippocampus (*78*), hence underscoring the translational relevance of these anatomical alterations. The role of Ube3a in neuronal morphology growth and maturation has been largely investigated (*79*), but the exact relationship between *Ube3a* gene dosage and large-scale brain anatomy remains elusive. Our findings of sex-invariant anatomical changes, but sex-specific connectivity alterations, in Ube3a2X mice suggest that *Ube3a* overdosage may affect brain connectivity and anatomy through distinct etiopathological cascades.

Because of the pleiotropic influence of *Ube3a* on multiple molecular and transcriptional pathways, ranging from protein degradation to transcriptional effects on multiple genes, our results cannot be unambiguously attributed to a specific function of Ube3a. Mechanistic inferences are further compounded by uncertainty regarding the ligase activity of the C-terminal flagged Ube3a copies overexpressed in our mouse model (*27*): While in vitro studies showed loss of ligase function upon C-terminal Ube3a tagging (*40*, *80*–*83*), in vivo investigations revealed largely increased ubiquitination levels in brain lysates from Ube3a2X mice (*32*) and comparable behavioral phenotypes when C- or N-terminal–tagged Ube3a is overexpressed in this model (*44*). Because AS reflects impaired Ube3a ligase function (*84*), our finding that the transcriptional profile of Ube3a2X mice exhibits robust overlap with 15qdup, but not with AS, argues against a prominent loss of ligase activity in our model and suggests that that the reported phenotypes may primarily reflect Ube3a-mediated transcriptional dysregulation. This hypothesis is consistent with Ube3a’s transcriptional effects being independent of its ligase activity (*33*, *34*) and our observations of robust multiomic sex biasing influences, an effect that could reflect transcriptional coactivation with steroid hormone receptors (*35*). Our finding that cortical transcriptome in Ube3a2X mice overlaps with gene lists related to steroid hormone receptor–relevant mechanisms lends further indirect support to this notion. Collectively, these observations suggest that *Ube3a* overexpression may affect gene networks and systems under the influence of diverse sex-relevant mechanisms, including the X chromosome, effects of genes sensitive to steroid hormone influence, downstream targets of steroid hormone receptors, as well as genes that are sex-differentially targeted by transcription factors. However, a putative downstream involvement of the ligase activity of Ube3a may also contribute to the sex-specific phenotypes we observed, since Ube3a has also been shown to ubiquitinate ER-α to target the receptor to proteasomal degradation (*85*). Future investigations of sex bias in other rodent models of *Ube3a* overdosage (*45*, *76*, *77*) may help corroborate or disprove these postulated mechanism.

The sex-specific effects observed in this study are congruent with both female-protective and male-enhancing risk explanations (*4*, *86*). *Ube3a* overexpression causes sex-specific transcriptional effects in many autism-associated genes, including *fmr1*, *scn2a*, *pten*, *cul3*, *mef2c*, *sox5*, *gabrb2*, and *gabrb3* (*62*). Many of these genes tend to be associated with autism via rare loss-of-function de novo mutations. Congruent with an interpretation of male-biased risk and female protection, these and other genes were underexpressed in Ube3a2X males and overexpressed in females. Behaviorally, the observation that male Ube3a2X mice showed increased stereotyped behaviors, while no differences were apparent in females, is broadly consistent with a possible female protection and male-potentiated genetic risk. It should, however, be noted that connectivity alterations in hypothalamic and motor-sensory areas were observed in female Ube3a2X mice. The possibility that this endophenotype is not compensatory, but instead, the expression of a distinct etiopathological signature cannot be entirely ruled out. This notion would be consistent with emerging evidence that some of the autism-associated genes that are dysregulated in our model (e.g., *Shank3*, *Tsc2*, *Mef2c*, or *Fmr1*) can lead to pathological cascades of translational relevance when they are either under- or overexpressed during development (*87*–*89*).

Our findings also indicate that *Ube3a* overdosage results in sex-specific dysregulation of processes and pathways on which diverse autism-associated genetic influences have been theorized to converge (e.g., synaptic dysregulation, aberrant transcription and translation/protein synthesis, and altered PI3K-AKT-mTOR signaling) (*62*–*64*). Diverse cell types theorized to be important in autism (e.g., excitatory and inhibitory neurons and glia cells) and that are affected by sex-relevant mechanisms are also affected differentially by M+F− and M−F+ gene sets (*7*, *10*, *36*–*39*, *90*, *91*). Thus, over and above providing a sex-specific influence on key autism-associated genes, *Ube3a* overdosage may be changing these emergent processes/pathways and cell types in males versus females to confer heightened male-risk and female protection. Future investigations, including cell type–specific overdosing of *Ube3a*, are required to elucidate the developmental and circuit mechanisms produced by Ube3a2X overexpression, and their possible relevance to 15qdup.

In conclusion, our data reveal robust sex-biasing effects on connectomics, repetitive behavior, and transcriptomic organization in mice with extra copies of *Ube3a*. These results suggest that *Ube3a* can critically contribute to sex bias in neurodevelopmental conditions like autism via influence on sex-relevant mechanisms, diverse neuronal and glial cell types, and important final common pathways that alter synaptic organization, transcription, translation, and other key signaling pathways (e.g., PI3K-AKT-mTOR).

## MATERIALS AND METHODS

### Ethical statement

Animal studies were conducted in accordance with the Italian law (DL 26/2014, EU 63/2010, Ministero della Sanità, Roma) and the recommendations in the *Guide for the Care and Use of Laboratory Animals* of the National Institutes of Health. Animal research protocols were also reviewed and approved by the Animal Care Committee of the University of Trento, Istituto Italiano di Tecnologia, and the Italian Ministry of Health (authorization no. 560/16). All surgical procedures were performed under anesthesia.

### Animal breeding and experimental cohorts

Mice were housed under controlled temperature (21 ± 1°C) and humidity (60 ± 10%). Food and water were provided ad libitum. Cages were equipped with high-quality wood shavings (trunk wood) and various types of enrichment material, including cardboard tunnels and housing, chewable wood sticks, and short-fiber cotton as substrate for nest construction.

Generation of Ube3a2X mice (FVB/NJ background) was previously described in (*27*). Mice were purchased from Jackson Laboratory, stock no: 019730. All experiments were performed on adult homozygous mice (*27*), which harbor two copies of *Ube3a* transgene independently from parental origin (Ube3a2X). Genotype was verified by quantitative PCR. WT adult littermate mice served as controls. Heterozygous females and males were used for mating. The average number of pups per litter was typically high (around 13 pups on average), and no overt deficit in maternal behavior was observed with genetically modified dams. Ratio of pups born/survived was around 1. All experimental female mice were virgin at the time of tests, and similarly, all the males used for testing had not previously mated. We report below the animal cohorts used in our study.

rsfMRI and structural MRI were performed on the same four cohorts of adult mice, ranging from 10 to 30 weeks of age: WT control females (*n* = 10), WT control males (*n* = 10), Ube3a2X females (*n* = 10), and Ube3a2X males (*n* = 10). Open-field, three-chamber test and rotarod were performed in this order, on the same four cohorts of adult mice (16 to 32 weeks of age): WT control females (*n* = 16), WT control males (*n* = 14), Ube3a2X females (*n* = 12), and Ube3a2X males (*n* = 14). Grooming scoring was performed on four separate cohorts of mice (10 to 30 weeks of age): WT control females (*n* = 9), WT control males (*n* = 9), Ube3a2X females (*n* = 10), and Ube3a2X males (*n* = 10). Habituation/dishabituation social interaction test was performed on four separate cohorts of mice, 12 to 46 weeks of age: WT control females (*n* = 13), WT control males (*n* = 12), Ube3a2X females (*n* = 8), and Ube3a2X males (*n* = 10). RNA-seq experiments were carried out on *n* = 4 WT control females, *n* = 4 WT control males and on *n* = 5 Ube3a2X females and *n* = 5 Ube3a2X males, 10 to 25 weeks of age. qRT-PCR–based validation of RNA-seq top hits and Western blot analysis were performed on four cohorts of mice, 8 to 32 weeks of age: WT control females (*n* = 3), WT control males (*n* = 4), Ube3a2X females (*n* = 5), and Ube3a2X males (*n* = 4).

qRT-PCR analysis of candidate genes was also carried out on FVB mice receiving a perinatal (PND1) injection of AAV-PHP.B-hSyn-hUBE3At (*57*) or a yellow fluorescent protein (YFP)–transducing vector (control group). We chose to conduct these injections in FVB mice as these animals reconstitute the genetic background of the Ube3a2X mouse line. Here, we used four separate cohorts of mice, 8 to 9 weeks of age: *n* = 6 male YFP-injected, *n* = 6 male hUbe3a-injected, *n* = 6 female YFP-injected, and *n* = 5 female hUbe3a-injected mice.

### ICV injection

At PND1, we performed ICV injection of 1 μl of the AAV-PHP.B-hSyn-hUBE3At vector (titer = 1.6 × 10^14^ viral genomes/ml, courtesy of B. J. Philpot), which enables the overexpression of human Ube3a in the mouse brain (*57*). Control mice received a 1-μl injection of AAVPHP.eB-hSyn-YFP [titer = 1.8 × 10^13^ genome copies/ml, Addgene #117382 (*92*)]. The procedure involved cryoanesthesia of pups on wet ice for 3 min. Pups were then placed onto a cooled neonatal stage for surgery. ICV injection was carried out using the following coordinates: lambda (*X*, *Y*, *Z*) = (1, ±0.3, −2.0) mm. A 35-gauge needle (Nanofil 35-gauge beveled) mounted on a 10-μl Nanofil was used to bilaterally deliver 1 μl of viral suspension. After the surgery, pups were placed on a heating pad with nesting material before being returned to the home cage with the mother. ICV-injected mice were then sacrificed at PND60 with a lethal dose of anesthesia for brain extraction.

### Resting-state fMRI

rsfMRI data were acquired as previously described (*47*, *93*, *94*). Briefly, animals were anaesthetized with isoflurane (5% induction), intubated, and artificially ventilated (2% maintenance during surgery). The left femoral artery was cannulated for continuous blood pressure monitoring. After surgery, isoflurane was replaced with halothane (0.7%) to obtain light sedation. Functional data acquisition started 45 min after isoflurane cessation.

Data were acquired with a 7-T MRI scanner (Bruker) as previously described (*95*, *96*), using a 72-mm birdcage transmit coil and a 4-channel solenoid coil for signal reception. Co-centered single-shot rsfMRI time series were acquired using an echo planar imaging (EPI) sequence with the following parameters: repetition time (TR)/echo time (TE) of 1000/15 ms, flip angle of 30°, matrix of 100 × 100, field of view of 2.3 × 2.3 cm, 18 coronal slices, slice thickness of 600 μm for 1920 volumes (total duration 32 min).

Mean arterial blood pressure (MABP) was recorded throughout the imaging sessions (fig. S8, A to C). Ube3a2X had slightly lower MABP than control mice (two-way ANOVA, genotype effect, *P* < 0.05; fig. S8C), but values were well within the autoregulation window within which changes in peripheral blood pressure do not result in fMRI blood oxygen level–dependent (BOLD) changes (*97*). In keeping with a negligible contribution of genotype-dependent MABP changes to our findings, we did not find any correlation between fMRI global connectivity and MABP in areas exhibiting sex-specific differences such as the PFC (*r* = −0.07, *P* = 0.67; fig. S8D). Arterial blood gas levels [partial pressure of CO_2_ (*P*co_2_) and partial pressure of oxygen (*P*o_2_)] were measured at the end of the acquisitions to ensure effectiveness of artificial ventilation. All mice had values within physiological range (*P*co_2_ < 42, *P*o_2_ > 90 mmHg). Analysis of body weight revealed an effect of sex (*P* < 0.01, two-way ANOVA), but no genotype or sex*genotype interactions (*P* > 0.37, all tests; fig. S8E).

### rsfMRI connectivity mapping

Raw rsfMRI data were preprocessed as previously described (*47*, *96*, *98*). The initial 50 volumes of the time series were removed to allow for signal equilibration. Data were then despiked, motion-corrected, and spatially registered to a common reference mouse brain template. Motion traces of head realignment parameters (three translations + three rotations) and mean ventricular signal (corresponding to the averaged BOLD signal within a reference ventricular mask) were regressed out from each time course. All rsfMRI time series were also spatially smoothed (full width at half maximum of 0.6 mm) and band-pass–filtered to a frequency window of 0.01 to 0.1 Hz.

To obtain an unbiased identification of the brain regions exhibiting alterations in functional connectivity, we calculated global and local fMRI connectivity maps for all mice. Global fMRI connectivity is a graph-based metric that defines connectivity as the mean temporal correlation between a given voxel and all other voxels within the brain. Local connectivity strength was mapped by limiting this measurement to connections within a 0.6252-mm (six voxels in-plane) sphere around each voxel (*95*, *99*). rsfMRI connectivity was also probed using a seed-based approach (*96*, *100*). A 3 × 3 × 1 seed region was selected to cover the areas of interest, and volume of interest (VOI)–to-seed correlations were computed. Pearson’s correlation scores were first transformed to *z* scores using Fisher’s *r*-to-*z* transform and then averaged to yield the final connectivity scores.

Voxel-wise intergroup differences in global and local connectivity and seed-based maps were assessed using a linear model including sex, genotype, and sex*genotype as factors (lm function in R studio). Data were imported into R using the oro.nifti package. The obtained *t* score maps were (FWER) cluster-corrected using a cluster threshold of *P* = 0.05.

### Structural MRI

To locate and quantify GM changes in Ube3a2X mice, we performed postmortem voxel-based morphometry (VBM) as previously described (*101*). Briefly, mice were deeply anesthetized with 5% isoflurane, and their brains were perfused via cardiac perfusion of 4% paraformaldehyde added with a gadolinium chelate to shorten longitudinal relaxation times. High-resolution morpho-anatomical T2-weighted MRI of mouse brains was performed using a 72-mm birdcage transmit coil, a custom-built saddle-shaped solenoid coil for signal reception. For each session, high-resolution morpho-anatomical images were acquired with the following imaging parameters: FLASH 3D sequence with TR = 17 ms, TE = 10 ms, α = 30°, matrix size of 260 × 180 × 180, FOV of 1.83 × 1.26 × 1.26 cm, and voxel size of 70 μm (isotropic).

Morpho-anatomical differences in local GM volumes were mapped using a registration-based VBM procedure (*47*, *101*, *102*). Specifically, high-resolution T2-weighted images were corrected for intensity nonuniformity, skull-stripped, and spatially normalized to a study-based template using affine and diffeomorphic registrations. Registered images were segmented to calculate tissue probability maps. The separation of the different tissues was improved by initializing the process with the probability maps of the study-based template previously segmented. The Jacobian determinants of the deformation field were extracted and applied to modulate the GM probability maps calculated during the segmentation. This procedure allowed the analysis of GM probability maps in terms of local volumetric variation instead of tissue density. Brains were also normalized by the total intracranial volume to further eliminate overall brain volume variations and smoothed using a Gaussian kernel of 3-voxel width. To quantify volumetric changes identified with VBM, we used preprocessed images to independently calculate the size of neuroanatomical areas via volumetric anatomical labelling (*101*).

### Behavioral tests

#### Open-field test

To test spontaneous locomotion, experimental mice were individually placed in an open-field arena (40 cm × 40 cm × 40 cm) and let free to explore for 10 min. The walls of the arena were smooth and gray-colored. Sessions were recorded and mice were automatically tracked using EthoVisionXT (Noldus). Locomotor activity was measured as total distance and mean velocity. In addition, the proportion of time spent in the center of the arena and outer zones was analyzed to estimate the level of anxiety. The number of full body rotations and time spent wall rearing were also measured.

#### Spontaneous self-grooming

Experimental mice were individually placed in an open-field arena and allowed to explore. Following a 10-min habituation period, the cumulative time spent self-grooming was scored for 10 min as indicator of stereotypic behavior as in (*103*, *104*).

#### Rotarod

The rotarod test is widely used for the evaluation and assessment of locomotor activity and motor coordination in rodents (*105*). Mice were pretrained on the rotarod apparatus for 3 days before the test. This habituation process involved performing at a consistent speed of 4 rpm for 5 min. On the fourth day, mice were tested for 5 min in three different trials. During each trial, the rotating rod accelerated from 4 to 64 rpm. Mice had 5 min of rest between each trial. The total time that the mice spent on the rotating rod was measured. The trials ended when the mice fell down or three consecutive full rotations were observed.

#### Habituation/dishabituation social interaction test

Animals were tested as previously described (*56*). Experimental mice were individually placed in a testing cage [GR900 Tecniplast cages (904 cm^2^)], lightly illuminated (5 ± 1 lux), 1 hour before the test. A matching stimulus mouse (same sex, same strain, and same age) was introduced into the testing cage for a 1-min interaction. At the end of the trial, the stimulus mouse was removed for 3 min. This sequence was repeated for four trials. Finally, experimental mouse was tested in a fifth 1-min dishabituation trial where a new stimulus mouse was introduced in the testing cage. Time spent interacting (sum of nose-to-nose sniffing, anogenital sniffing, and following) was scored across trials by an experimenter blind to genotypes.

#### Three-chamber social interaction test

Each mouse’s preference for a conspecific over an inanimate object (sociability), as well as its preference for a stranger mouse over a familiar mouse (social novelty) was assessed using previously established three-chamber assay (*106*). During the sociability phase, a stranger mouse was placed in one chamber inside a wire cup that allowed nose contact. An identical novel cup was placed in the opposite side chamber. Video monitoring of the test mouse’s exploration of the apparatus was carried on for 10 min. Next, in the social novelty phase, the test mouse was re-exposed for 10 min to the initial stranger (now familiar) mouse, as well as to a novel stranger mouse placed inside the second wire cup, in the opposite chamber. In addition to the automatically tallied time spent in each chamber, we manually scored the time spent sniffing the cups and the stimulus mice, as well time spent sniffing the empty cup. A sociability index was calculated as the time spent sniffing the mouse cup minus time sniffing the empty cup divided by the total sniffing time ($\frac{{\text{Time}}_{\text{social}}-{\text{Time}}_{\text{empty}}}{{\text{Time}}_{\text{social}}+{\text{Time}}_{\text{empty}}}$). Accordingly, the sociability index during the social novelty phase (novelty index) was calculated as the time spent sniffing the novel mouse cup minus time spent sniffing the familiar mouse cup divided by the total sniffing time ($\frac{{\text{Time}}_{\text{novel}}-{\text{Time}}_{\text{familiar}}}{{\text{Time}}_{\text{novel}}+{\text{Time}}_{\text{familiar}}}$).

#### RNA-seq and preprocessing

Mice of both genotypes and sexes were sacrificed by cervical dislocation, and PFC and hypothalamus were rapidly identified according to the Allen Mouse Brain Atlas (www.brain-map.org) and dissected. The samples used for RNA-seq experiment and a complete list of quality control parameters are deposited in Gene Expression Omnibus (GEO) (GSE217420). Brains were rotated, and the exposed hypothalamus was excised with surgical tweezers. To collect PFC tissue, explanted brains were placed in an adult mouse brain matrix (Agnthos, Sweden). Two coronal sections at the level of the PFC for each mouse brain were collected (*107*). The sections (1 mm thick) were cut with scalpel blades and immediately put on a semi-frozen glass slide. Tissue from PFC was obtained by micropunches of 0.5 mm. One micropunch for each hemisphere/section was collected, for a total of four micropunches per brain. Following tissue collection, samples were frozen with dry ice and stored at −80°C until RNA extraction.

#### RNA extraction and library preparation

All procedures were conducted in ribonuclease (RNase)–free conditions. On the day of RNA extraction, the hypothalamus and PFC tissues were disrupted and homogenized for 3 min using motor-driven grinders. Total RNA isolation was performed using RNeasy Mini Kit and RNeasy Micro Kit (Qiagen), respectively, following the manufacturer’s instructions. RNA concentration was evaluated using Qubit RNA BR Assay Kit (Life Technologies). RNA purity was assessed by determining ultraviolet (UV) 260/280 and 260/230 absorbance ratios using a NanoDrop ND-1000 spectrophotometer (Thermo Fisher Scientific). RNA quality was evaluated by measuring the RNA integrity number (RIN) using an Agilent RNA 6000 Nano Kit with an Agilent 2100 Bioanalyzer (Agilent Technologies, Santa Clara, CA, USA) according to the manufacturer’s instructions. All samples had RIN of >6.8 (see GEO repository, GSE217420). Libraries for RNA-seq were prepared using the paired-end TruSeq Stranded mRNA Sample Preparation kit (Illumina, San Diego, Ca, USA) according to the manufacturer’s instructions. For each sample of hypothalamus and PFC, 1000 ng and 500 ng were used as input quantity, respectively. The libraries were prepared in one batch using NovaSeq 6000 S2 Reagent Kit (200 cycles) at an average read depth of 100 million paired-end reads. Libraries were 0.85 nM in a volume of 150 μl and loaded on an Illumina NovaSeq 6000 System (Istituto Italiano di Tecnologia, Center for Human Technologies, Genomic Unit, Genova, Italy).

Raw reads were aligned to the mm10 genome (GRCm38 primary assembly obtained from the Gencode website) using the STAR aligner and counted with featureCounts using the gene annotation Gencode v24. Picard tools (http://broadinstitute.github.io/picard/) functions were used to quantify sequencing-related variables. Low-read genes were removed if they had less than 100 reads in two or more samples. Variance filtering was used to filter out genes in the bottom 15%-tile ranked by variance. In PFC samples, this filtering resulted in 12,294 genes being retained for further analysis, while 13,727 genes were retained for Hypo samples. Normalization for library size was implemented with the calcNormFactors function in the edgeR R library, using the trimmed mean of M values (TMM) method (*108*). The voom function from the limma library in R (*109*) was then used to transform the data to log counts per million and estimate precision weights to incorporate in the linear modeling of differential expression (DE). Finally, surrogate variable analysis (SVA) was used to estimate artifact-related variables (*110*). This was achieved by constructing a model of the known variables to account for (i.e., sex, genotype, sex*genotype interaction, and RIN) and then having SVA estimate surrogate variables (SVs) from the error term of the model. The number of SVs estimated was 2 for both PFC and Hypo. To better understand how these SVs account for known sequencing-related artifact variables, we first ran a principal components analysis (PCA) on the Picard variables of percent coding bases, percent utr bases, percent intronic bases, percent intergenic bases, median CV coverage, median 5′ to 3′ bias, aligned reads, and AT dropout. This gave an orthogonal summary of the artifact-related variables, and we then analyzed these PCs for correlations with the SVs. We find that both SVs are highly correlated with many of the sequencing-related artifact PCs (fig. S9), indicating that substantial sequencing-related artifact is accounted for parsimoniously with two SVs. Furthermore, to see how these SVs correlate with principal axes of variance in the expression data, we correlated SVs with the first 10 principal components of the expression data. Here again, the SVs known to be relevant for sequencing-related artifact are highly correlated with the first PCs in the expression data (fig. S9), indicating that without removing these artifact-related structured noise variables, they would swamp a large proportion of the variance in the expression data. Therefore, we used these SVs in the linear modeling for DE to account for and remove variance associated with these artifact-related SVs.

#### DE analysis

DE analysis was achieved using functions for linear modeling in the limma library in R. DE analysis examined specific contrasts for the sex*genotype interaction as well as main effects of sex and group, respectively. The linear model included RIN and SVs as covariates and incorporated the precision weights estimated by voom:

$$\text{logcpm}\sim {\text{group}}^{*}\text{sex}+\text{RIN}+\text{sv}1+\text{sv}2$$

where logcpm is the log counts per million, group is the genotype, RIN is the RNA integrity, and sv is the surrogate variables identified by SVA.

DE models were computed separated for PFC and Hypo samples. Genes that pass FDR *q* < 0.05 (*111*) were considered DE.

#### Enrichment analysis

To annotate DE gene sets for Kyoto Encyclopedia of Genes and Genomes (KEGG) pathways and mouse brain cell types, we used the enrichR library in R (https://maayanlab.cloud/Enrichr/) (*112*, *113*). Mouse brain cell types were based on data cortex and hippocampal tissue samples in the Allen Institute 10× single-cell RNA-seq (scRNA-seq) dataset (*65*) (https://portal.brain-map.org/atlases-and-data/rnaseq). For GO BP enrichment analysis, we used the GeneWalk library in Python (https://github.com/churchmanlab/genewalk) (*114*). With custom gene lists of relevance to autism- and sex-related genomic mechanisms, we ran additional gene set enrichment tests with DE gene sets. These tests were run using custom code for running gene set enrichment analysis that computes enrichment ORs and *P* values based on the hypergeometric distribution. For these tests, we used a background total equivalent to the total number of genes analyzed after filtering in the main gene expression analyses (e.g., 12,294 for PFC and 13,727 for Hypo). Because autism- and sex-related gene lists are based on human gene symbols, we first converted mouse gene IDs into human gene homologs and then ran all enrichment tests. All results of these enrichment tests were thresholded at FDR *q* < 0.05. The diagnostic gene lists were a curated list of high-impact autism-associated genes from SFARI Gene (https://gene.sfari.org; downloaded January 2021; the SFARI gene list includes all genes from all categories 1, 2, 3, and S) and DE genes from postmortem cortical tissue in autism, schizophrenia, bipolar disorder (*59*), and duplication 15q syndrome (*43*) and from iPSC-derived neurons from dup15q and AS patients (*60*). Sex-related gene lists included downstream targets of the androgen (*36*) and estrogen receptors (*115*), and genes that are sex-differentially targeted by transcription factors (*116*). We also ran similar enrichment analyses for chromosomes to test if DE gene sets were significantly enriched for genes located on specific chromosomes (e.g., X chromosome). The background list of genes is reported in table S5. All the lists of genes used in the enrichment tests are reported in table S6.

#### PPI analysis

To better understand how key DE genes may work together in specific ASD-relevant systems biological processes, we used STRING-DB (https://string-db.org/) to conduct a PPI analysis whereby the input gene list was *Ube3a*, steroid hormone receptors (ar, esr1, esr2), and PFC sex-by-genotype DE genes that were annotated as significantly enriched in GeneWalk GO biological processes of relevance to autism (e.g., synaptic, transcription, translation, mTOR, and ERK signaling pathways) or of relevance to steroid hormone receptor signaling. This analysis was done using the human gene homologs of the mouse DE genes and applies standard STRING defaults in the analysis (i.e., full network type, confidence level = 0.4). The resulting PPI network plot is then colored with a data-driven *k*-means clustering with *k* = 3 to visually demarcate proteins that cluster into largely synaptic, transcription/signaling, and translation sets.

#### Quantitative RT-PCR

Adult (12- to 16-week-old) Ube3a2X- and FVB-injected mice were euthanized, and their brains were promptly extracted, carefully dissecting either the entire cortex, or medial PFC, using forceps. The dissected samples were rapidly frozen in dry ice and stored at −80°C. Total RNA extraction from these samples was performed using the RNeasy Plus Mini Kit (Qiagen). Subsequently, the RNA underwent reverse transcription into cDNA using the SuperScript VILO cDNA Synthesis kit (Thermo Fisher Scientific) following the manufacturer’s instructions.

Subsequently, we carried out qRT-PCR in a Bio-Rad C1000 Thermal Cycler, using the PowerUp SYBR Green Master Mix (Applied Biosystems). To prevent the amplification of genomic DNA, primers (Sigma) were designed across different exons (refer to the list of primer sequences below). Expression analyses were executed using CFX3 Manager 3.0 software from Bio-Rad.

Mean cycle threshold (*C*_t_) values were determined from triplicate experiments for each gene of interest and the reference housekeeping gene, β-actin. These values underwent adjustment for PCR efficiency and inter-run calibration.

Statistical analyses were carried out using a two-way ANOVA followed by Tukey post hoc analysis, with a significance level set at *P* < 0.05.

The following primer sequences were used: Fmr1, 5′-GTGAGGGTGAGGATTGAGGC (forward) and 5′-TGAAACCACTAACACCCTCTGG-3′ (reverse); Ube3a, 5′-GGCGAGGACAGATCACCAG-3′ (forward) and 5′-AGGCCTCATTTCCACAGCC-3′ (reverse); Scn2a, 5′-ATGACCATGAGCAACCCTCC-3′ (forward) and 5′-CAAATTCTGTTACATACGCAAAGGT-3′ (reverse); Cul3, 5′-ACAGCATTTGGGACCTTCTGA-3′ (forward) and 5′-TCTTCTCGCACC TTATTTATGAGA-3′ (reverse); Gabrb3, 5′-AGGAAGGCTTTTCGGCATCT-3′ (forward) and 5′-GGGGTCGTTTACGCTCTGA-3′ (reverse); hUbe3a, 5′-GTTCCTCCAATTCACCACC-3′ (forward) and 5′-CACGTTGAAACAAGTGTGGG-3′ (reverse).

#### Western blot

PFC tissue samples from adult Ube3a2X mice (13 to 15 weeks old) were lysed using lysis buffer [10 mM tris (pH 7.4), 0.5% NP-40, 0.5% Triton X-100, 150 mM NaCl plus Protease and Phosphatase Inhibitor Cocktail Tablets, Roche) via mechanical homogenization. Protein quantification was carried out with Pierce BCA quantification kit following the manufacturer’s instructions (Thermo Fisher Scientific). Samples were boiled in Laemmli sample buffer containing 100 mM dithiothreitol (DTT) for 5 min at 95°C and loaded on SDS polyacrylamide gel electrophoresis (SDS-PAGE) using precast gels (Any kD, Bio-Rad) and then transferred to nitrocellulose membranes. Membranes were blocked for 1 hour in 5% (w/v) nonfat dry milk in TBS-T or 5% bovine serum albumin (BSA) in TBS-T (tris-buffered saline containing 0.01% Tween 20). Blots were probed with anti-UBE3A (BD 611416; 1:1000 in 5% BSA). After incubation with primary antibodies, membranes were washed three times with TBS-T (10 min each) and then probed with a 1:15,000 dilution of anti-mouse horseradish peroxidase (HRP) conjugated (Jackson ImmunoResearch 115-035-003) for 1 hour, at room temperature. After two washes with TBS-T and one with Milli-Q water, signals were revealed using the ECL Prime Western Blotting Detection Kit (GE Healthcare) and visualized with a ChemiDoc Imaging System (Bio-Rad). We obtained the final quantification of proteins detected by primary antibodies using densitometric analysis of the Western blots and normalizing the signal on the corresponding total protein lane [obtained by the enhanced tryptophan fluorescence technology of stain-free gels, Bio-Rad (*117*, *118*)]. Statistical analysis of Western blot data was performed by two-way ANOVA using the GraphPad Prism 8 software with a significance level set at *P* < 0.05.
