# Supplementary material for: Sex-biasing influence of autism-associated Ube3a gene overdosage at connectomic, behavioral, and transcriptomic levels
Source: Sci Adv. 2024 Jul 12;10(28):eadg1421. doi: 10.1126/sciadv.adg1421 (PMC11244557; doi:10.1126/sciadv.adg1421)
Supplement: Supplementary file 1 — Figs. S1 to S9 Legends for tables S1 to S6 [file sciadv.adg1421_sm.pdf]

Science Advances
AAAS

Supplementary Materials for

Sex-biasing influence of autism-associated *Ube3a* gene overdosage at connectomic, behavioral, and transcriptomic levels

Caterina Montani *et al.*

Corresponding author: Alessandro Gozzi, alessandro.gozzi@iit.it; Michael V. Lombardo, mvlombardo@gmail.com

Sci. Adv. **10**, eadg1421 (2024)
DOI: 10.1126/sciadv.adg1421

The PDF file includes:

Figs. S1 to S9
Legends for tables S1 to S6

Other Supplementary Material for this manuscript includes the following:

Tables S1 to S6

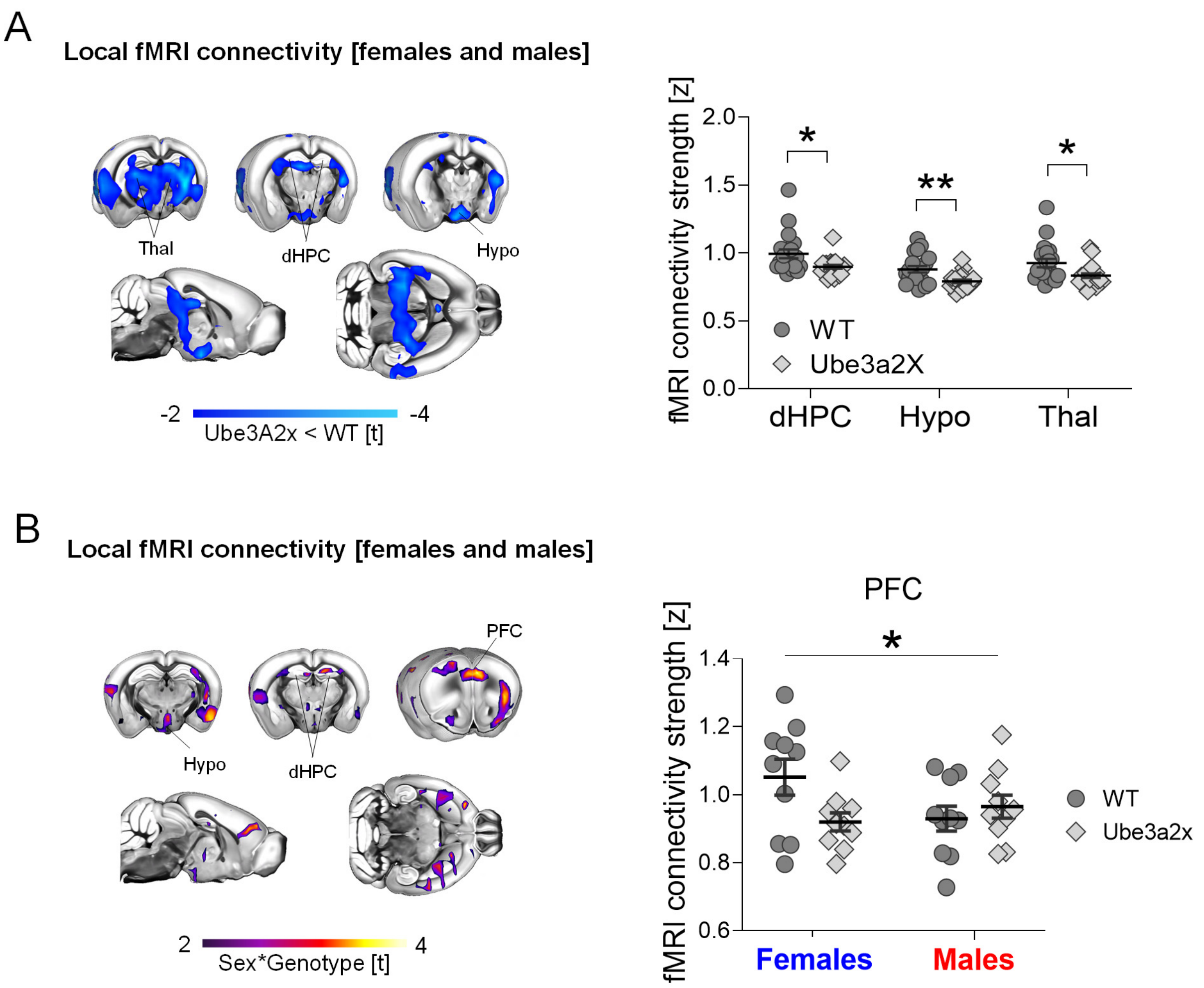

Supplementary Figure S1

Increased *Ube3a* dosage affects local fMRI connectivity in a sex-dependent manner. A) Intergroup contrast maps (left panel) showing reduced local fMRI connectivity strength in Ube3a2X animals (n = 20) compared to WT control (n = 20) littermates (both sexes, blue color indicates reduced connectivity, t-test, t > 2; FWE cluster-corrected). Panel on the right illustrates quantification of global fMRI connectivity strength in representative regions of interest (t-test, thalamus, t = 2.57, p = 0.015; dorsal hippocampus, t = 2.62, p = 0.014; hypothalamus, t = 3.25, p = 0.028). B) Contrast maps (left panel) showing areas exhibiting sex*genotype interactions in local fMRI connectivity strength (purple and yellow coloring, t > 2; FWE cluster-corrected). Panel on the right illustrates quantification sex*genotype interaction in representative regions of interest. (ANOVA interaction, F = 6.68, p = 0.019). dHPC, dorsal hippocampus; Hypo, Hypothalamus; PFC, Prefrontal Cortex, Thal, Thalamus. *p < 0.05, **p < 0.01. FWE: family-wise error. Error bars of the plots indicate SEM and each dot represents a mouse.

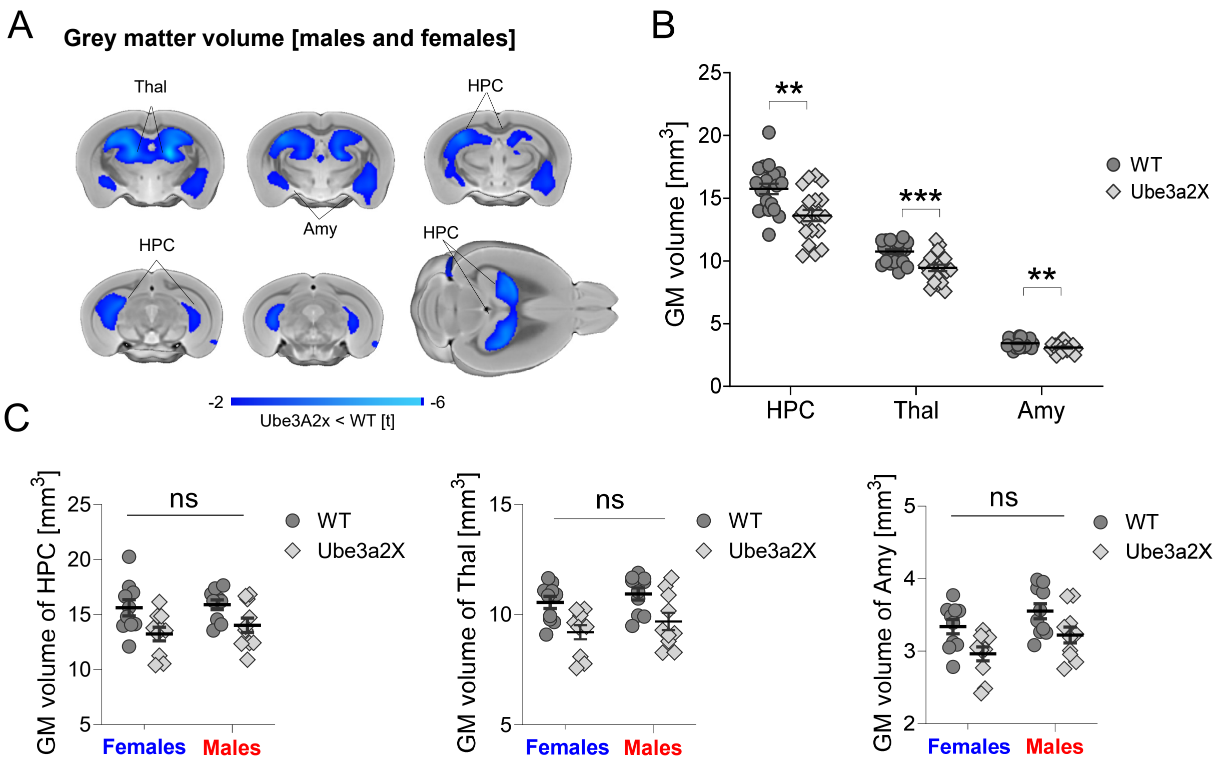

Supplementary Figure S2

Brain anatomy is not affected by *Ube3a* dosage in a sex-dependent manner. A) Structural MRI showing a reduction in gray matter volume in Ube3a2X mutants (n=20) compared to WT control (n=20) littermates (both sexes, t > 2; FWE cluster-corrected, blue coloring). **B)** Regional quantifications showing reduction of gray matter volume in hippocampus (t = 3.47, p = 0.001), thalamus (t = 4.097, p < 0.001) and amygdala (t = 3.29, p = 0.002) in Ube3a2X mutants compared to WT mice (both sexes). **C)** Sex*genotype interaction in gray matter volume was not significant ("ns") in all the quantified regions, including amygdala (ANOVA, sex*genotype interaction, F = 0.05, p = 0.82), thalamus (ANOVA, sex*genotype interaction, F = 0.025, p = 0.87) and hippocampus (ANOVA, sex*genotype interaction, F = 0.17, p = 0.68). The absence of significant sex*genotype interactions reflects concomitantly reduced GM volume in both Ube3a2X females (t-test, amygdala, t = 2.59, p = 0.027; thalamus, t = 3.60, p = 0.009; hippocampus, t = 2.71, p = 0.002) and Ube3a2X males (unpaired t-test, amygdala, t = 2.59, p = 0.057; thalamus, t = 2.78, p = 0.017; hippocampus, t = 2.13 , p = 0.08) compared to sex-matched WT control mice. Amy, Amygdala; Thal, Thalamus; Hypo, Hypothalamus; *p < 0.05, **p < 0.01; FWE, family-wise error; ns, non-significant; GM, gray matter, Error bars of the plots indicate SEM and each dot represents a mouse.

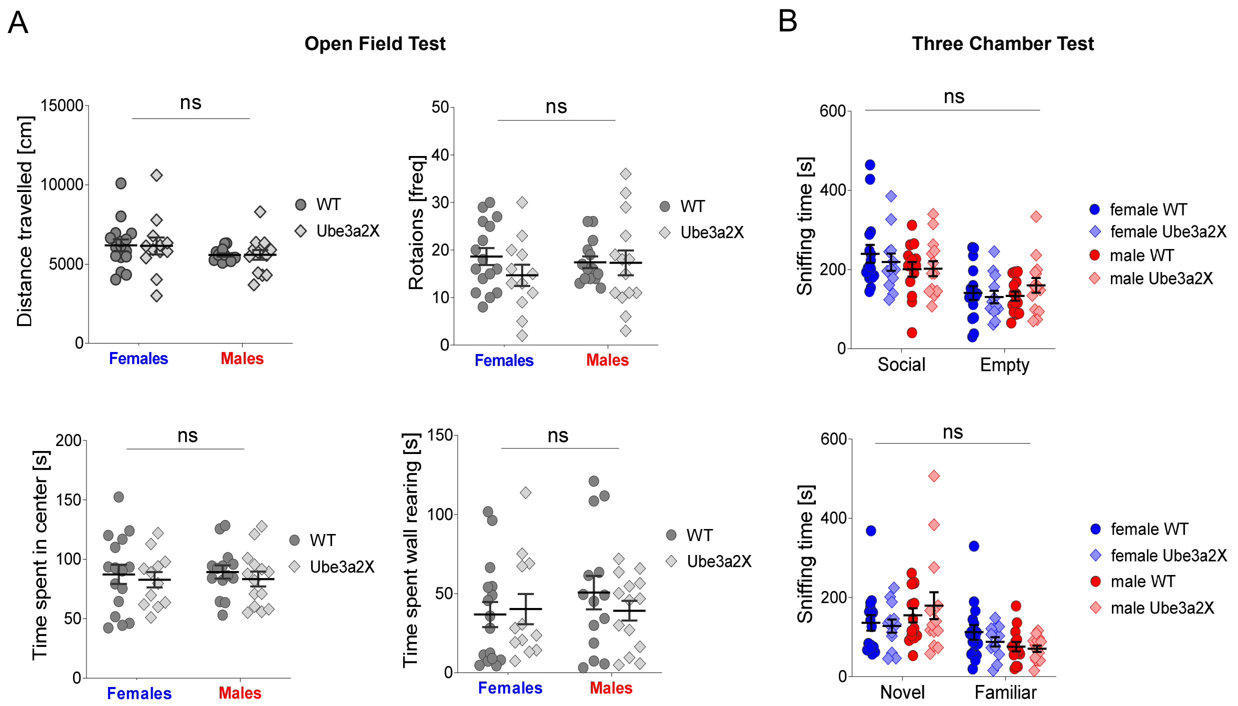

Supplementary Figure S3

Ube3a2X mice do not show gross alterations in social and anxiety-related behaviors. A) Results of open field test. Sex*genotype interaction was not significant ("ns") for travelled distance ($F = 0.005$, $p = 0.94$), frequency of rotations ($F = 0.89$, $p = 0.35$), total time spent in the center ($F = 0.011$, $p = 0.92$) and wall rearing ($F = 0.73$, $p = 0.39$, ANOVA, sex*genotype interaction). All four behaviors were unimpaired in Ube3a2X mutants (n = 27, n = 14 males and n = 13 females) compared to sex-matched WT littermates (n = 30, n = 14 males and n = 16 females) ($p > 0.20$, all behaviors). B) Three chamber test. The total time spent sniffing is reported for sociability (top panel) and the social novelty phase (bottom panel). Sex*genotype interaction in sociability (ANOVA, sex*genotype interaction, $F = 1.24$, $p = 0.31$) and preference for social novelty (ANOVA, sex*genotype interaction, $F = 0.39$, $p = 0.76$) were not significant ("ns"). Chamber effect was significant in both phases ($F = 20.71$, $p < 0.0001$, $F = 18.3$, $p < 0.0001$, respectively). ***$p < 0.001$. All plots report mean± SEM, on top of individual data points.

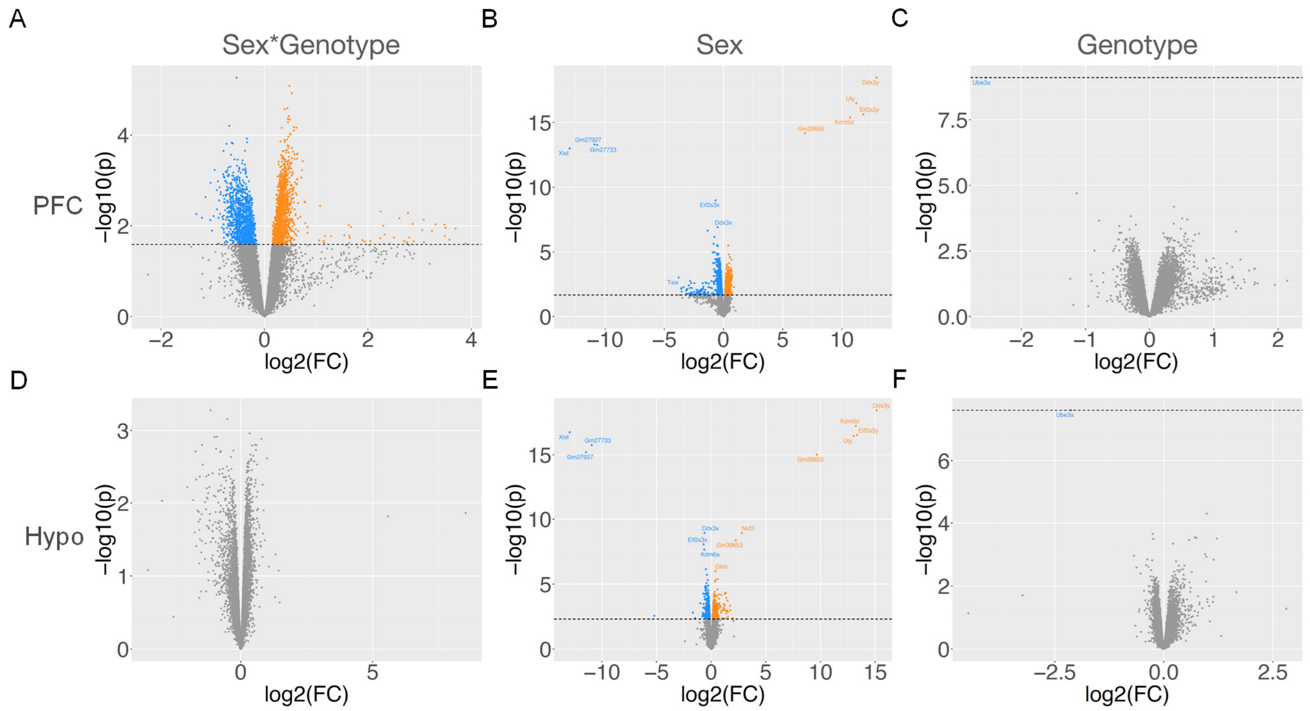

Supplementary Figure S4

Genes are dysregulated in a sex*genotype manner in the PFC. Rows indicate PFC data (top, **A-C**) or Hypo data (bottom, **D-F**). Volcano plots show log2 fold change (FC) on the x-axis and -log10 p-values on the y-axis for the effects of the sex*genotype interaction (left, **A, D**), main effect of sex (middle, **B, E**), and main effect of genotype (right, **C, F**). Genes colored in orange are the M-F+ genes, while genes colored in blue are M+F- genes. Genes depicted in gray fall below the horizontal dotted line indicating the FDR q<0.05 threshold, and are thus considered not differentially expressed.

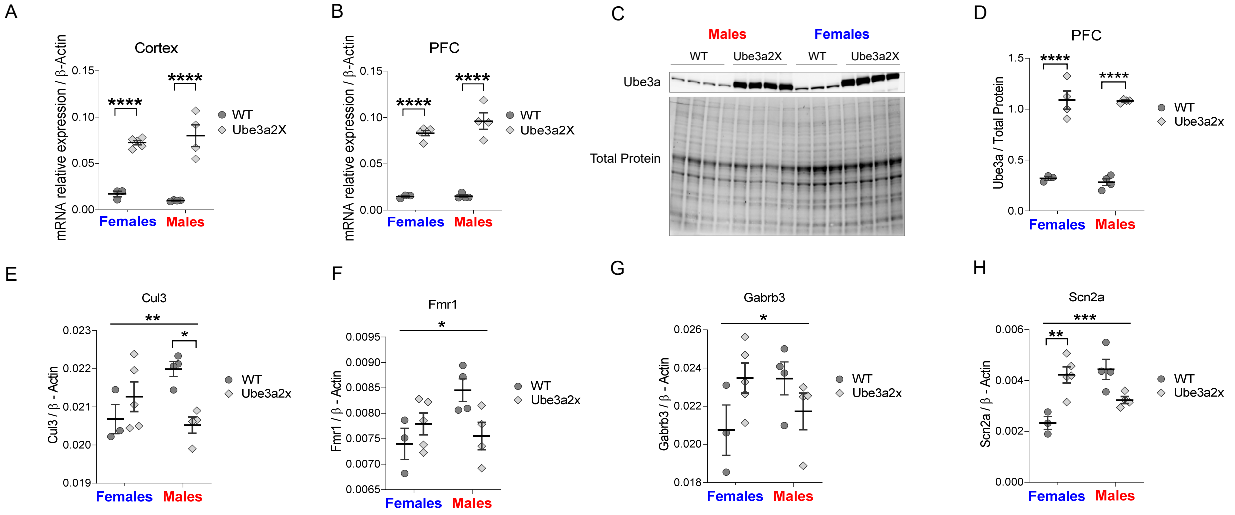

Supplementary Figure S5

qRT-PCR quantifications and Western blot analyses of Ube3a. Relative mRNA expression level as obtained by qRT-PCR performed on **A)** whole cerebral cortex (sex*genotype interaction, F = 1.36, p = 0.26, sex factor F = 0.0014, p = 0.97, genotype factor F = 100.2, p < 0.0001. Tukey's post hoc test for multiple comparisons, ****p < 0.0001), or **B)** PFC extracts of adult wild type (WT) and Ube3a2X male and female mice (sex*genotype interaction, F = 1.67, p = 0.22, sex factor F = 1.82, p = 0.20, genotype factor F = 229, p < 0.0001. Tukey's post hoc test for multiple comparisons ****p < 0.0001). Each dot indicates an individual animal. **C)** Western Blot analysis of Ube3a on PFC lysates from WT and Ube3a2X male and female animals. **D)** Relative protein expression level as obtained by Western Blot analysis on PFC extracts of adult wild type (WT) and Ube3a2X male and female mice (sex*genotype interaction, F = 0.075, p = 0.79, sex factor F = 0.19, p = 0.67, genotype factor F = 218.2, p < 0.0001. Sidak's post hoc test for multiple comparisons ****p < 0.0001). **Validation of RNAseq top hit genes via qRT-PCR. E-H)** Relative mRNA expression level as obtained by qRT-PCR performed on PFC extracts of adult WT and Ube3a2X male and female mice for the following genes: Cul3 (sex*genotype interaction, F = 9.95, p = 0.008), Fmr1 (sex*genotype interaction, F = 6.6, p = 0.02), Gabrb3 (sex*genotype interaction, F = 5.39, p = 0.039), and Scn2a (sex*genotype interaction, F = 24.7, p = 0.0003).

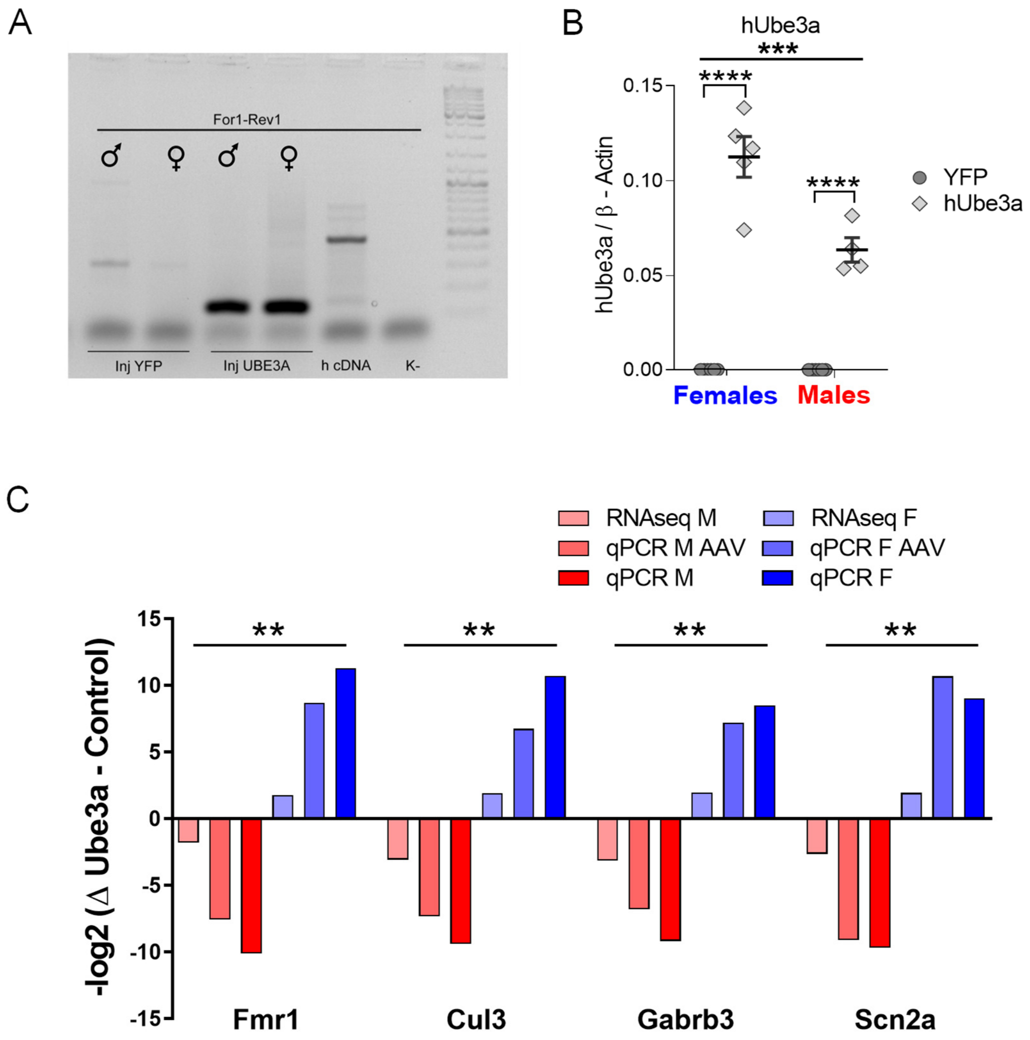

Supplementary Figure S6

hUbe3A overexpression in FVB mice. A) qRT-PCR showing expression of hUbe3a in male and female FVB mice injected with AAV-PHP.B-hSyn-hUBE3At vector (Inj UBE3A). AAVPHP.eB-hSyn-YFP (Inj YFP) was used as control. **B)** Relative mRNA expression level of hUbe3a, as obtained by qRT-PCR performed on PFC from hUbe3a injected animals and controls (ANOVA interaction, $F = 18.7$, $p = 0.0005$, sex factor $F = 18.7$, $p = 0.0005$, genotype factor $F = 240.5$, $p < 0.0001$. Tukey's post hoc test, ***$p < 0.001$, ****$p < 0.0001$). C) Plot showing the difference (Δ) in gene expression between Ube3a overexpressing animals (Ube3a2X and AAV-hUbe3a injected animals) and controls. Cul3, Fmr1, Gabrb3 and Scn2a showed the same directionality of the sex-specific effect across the 3 experiments: the first value plotted is mean gene expression obtained with RNAseq ("RNAseq Sex"); the second one is the result of qRT-PCR analysis performed on pooled samples from virally injected FVB mice (labelled "qPCR Sex AAV"); the third one is an independent validation of our prior RNAseq data in the mouse model that we obtained via qRT-PCR of candidate (ANOVA sex*gene interaction $F = 0.076$, $p = 0.97$, sex-factor $F = 65.7$, $p < 0.0001$, gene $F = 0.025$, $p = 0.99$. Sidak's post hoc test **$p < 0.01$).

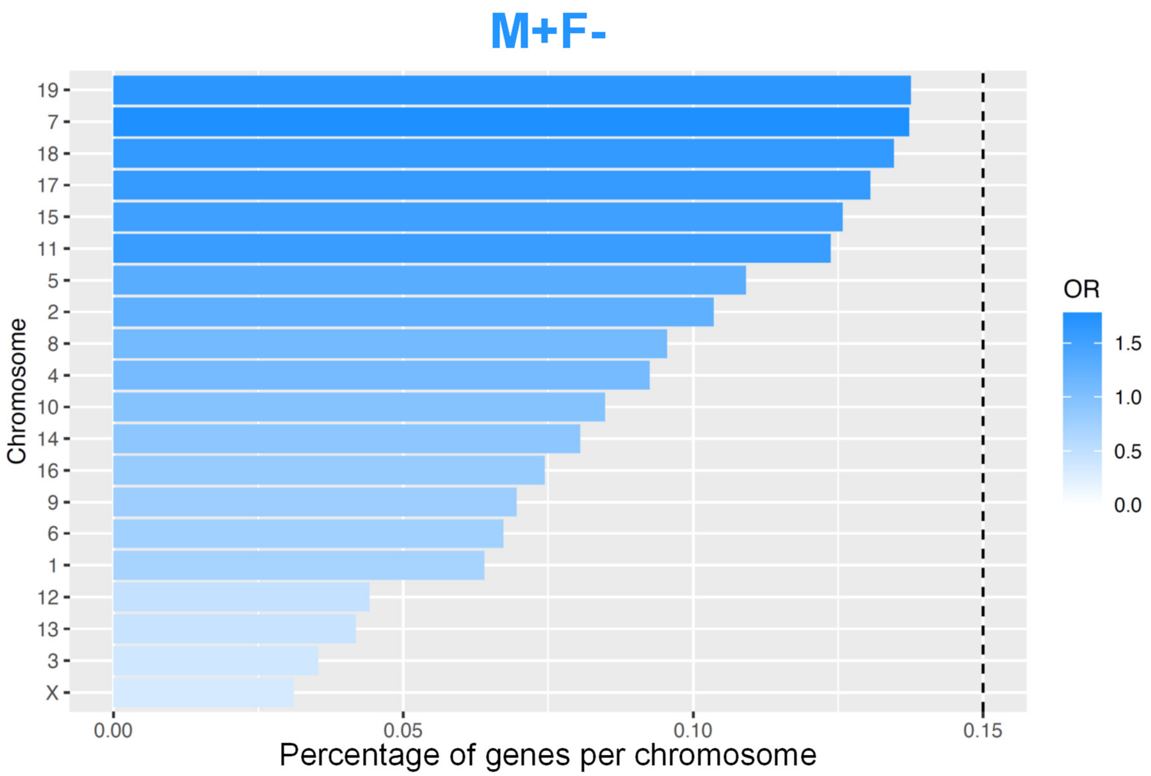

Supplementary Figure S7

DE+ genes are equally distributed across chromosomes. Plot showing the percentage of genes per each chromosome that are DE+. Color indicates the enrichment odds ratio. The vertical dotted line indicates the FDR q < 0.05 threshold.

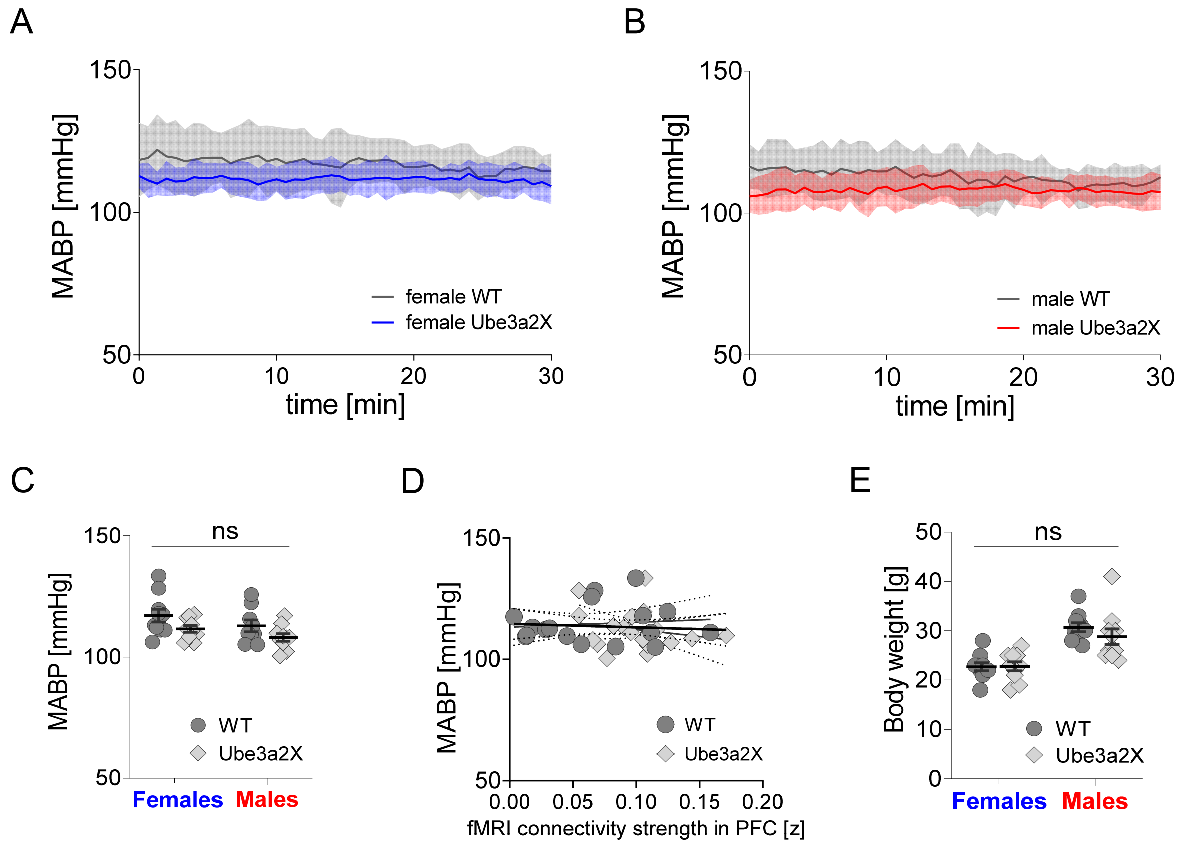

Supplementary Figure S8

Mean arterial blood pressure and body weight. A) Plot of mean arterial blood pressure in female Ube3a2X mice (n=10) and female WT littermates (n=10) during rsfMRI scanning. B) Plot of arterial blood pressure in male Ube3a2X mice (n=10) and male WT littermates (n=10) during rsfMRI scanning. C) Quantification of mean arterial blood pressure of Ube3a2X females (unpaired t-test, t = 1.91, p = 0.12) and Ube3a2X males (unpaired t-test, t = 1.62 p = 0.21) across the imaging time window. No significant sex*genotype interaction was observed in arterial blood pressure (ANOVA, sex*genotype interaction, F = 0.031, p = 0.86, "ns" in the plot). D) Lack of correlation between fMRI connectivity in a representative brain region (i.e. the prefrontal cortex) and mean arterial blood pressure (r = -0.07, p = 0.67). E) Body weight of Ube3a2X females (unpaired t-test, t = 0.06, p = 0.99) and Ube3a2X males (unpaired t-test, t = 1.22 p = 0.40) is comparable to that of sex-matched WT control mice. Sex*genotype interaction in body weight was not statistically significant (ANOVA, sex*genotype interaction, F = 0.82, p = 0.37, "ns" in the plot). ns, non-significant. Error bars of the plots indicate SEM.

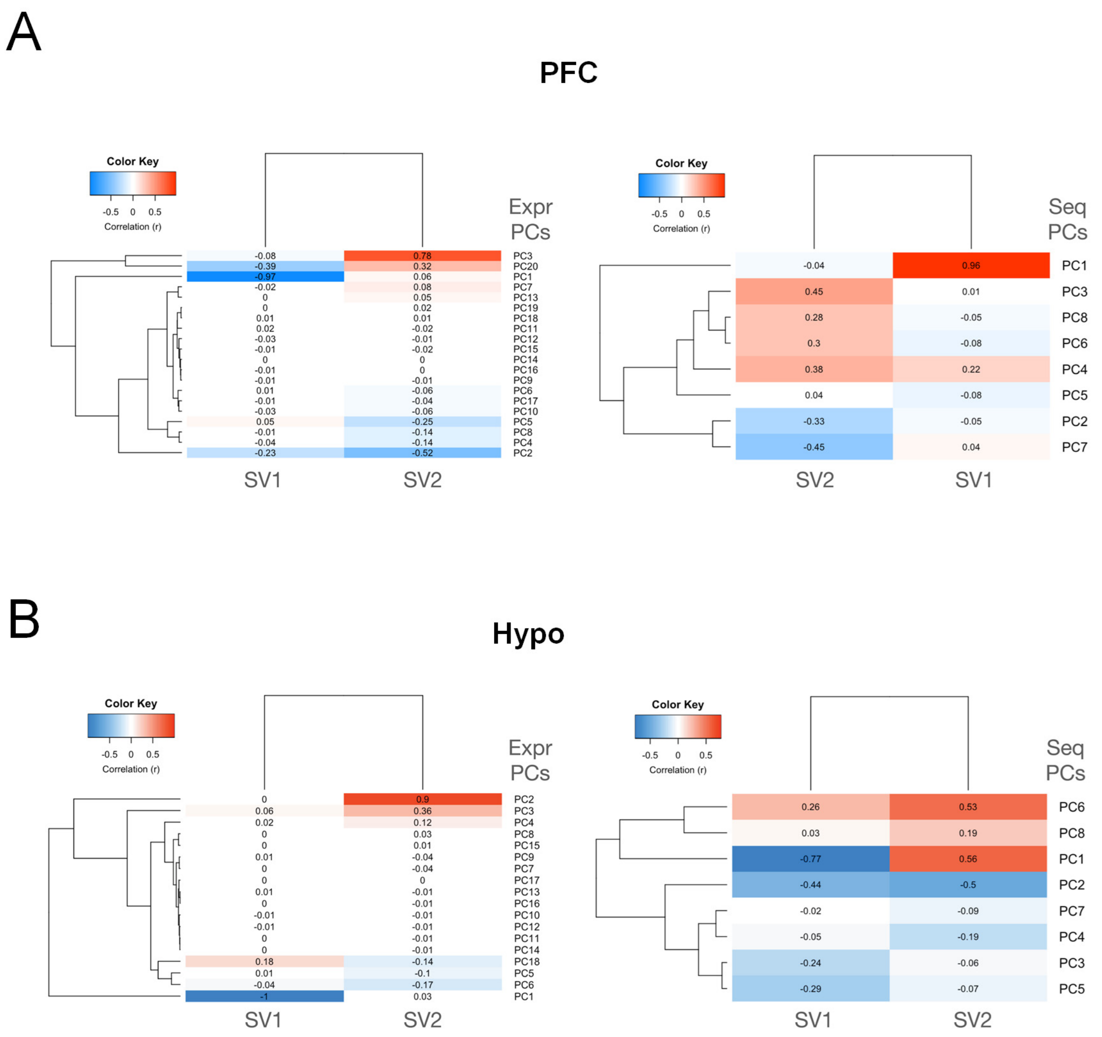

Supplementary Figure S9

Correlations between surrogate variables (columns SV1, SV2) and principal components (rows) derived from preprocessed gene expression data (left) or sequencing-related variables (right). Panel (A) shows these correlations for PFC data, while panel (B) shows the correlations from Hypo data.

Supplementary Table S1

Full list of DE genes in the PFC tested for sex*genotype interaction.

Supplementary Table S2

Full list of DE genes tested for the main effect of sex.

Supplementary Table S3

Full list of DE genes tested for the main effect of group (genotype).

Supplementary Table S4

Complete statistics of each of the gene lists used for the enrichment tests.

Supplementary Table S5

Background sets of all the genes tested for DE.

Supplementary Table S6

Lists of genes used for the enrichment tests.
